# Direct entry by RNase E is a major pathway for the degradation and processing of RNA in *Escherichia coli*

**DOI:** 10.1093/nar/gku808

**Published:** 2014-09-18

**Authors:** Justin E. Clarke, Louise Kime, David Romero A., Kenneth J. McDowall

**Affiliations:** Astbury Centre for Structural Molecular Biology, School of Molecular and Cellular Biology, Faculty of Biological Sciences, University of Leeds, Leeds, LS2 9JT, UK

## Abstract

*Escherichia coli* endoribonuclease E has a major influence on gene expression. It is essential for the maturation of ribosomal and transfer RNA as well as the rapid degradation of messenger RNA. The latter ensures that translation closely follows programming at the level of transcription. Recently, one of the hallmarks of RNase E, i.e. its ability to bind via a 5′-monophosphorylated end, was shown to be unnecessary for the initial cleavage of some polycistronic tRNA precursors. Here we show using RNA-seq analyses of ribonuclease-deficient strains *in vivo* and a 5′-sensor mutant of RNase E *in vitro* that, contrary to current models, 5′-monophosphate-independent, ‘direct entry’ cleavage is a major pathway for degrading and processing RNA. Moreover, we present further evidence that direct entry is facilitated by RNase E binding simultaneously to multiple unpaired regions. These simple requirements may maximize the rate of degradation and processing by permitting multiple sites to be surveyed directly without being constrained by 5′-end tethering. Cleavage was detected at a multitude of sites previously undescribed for RNase E, including ones that regulate the activity and specificity of ribosomes. A potentially broad role for RNase G, an RNase E paralogue, in the trimming of 5′-monophosphorylated ends was also revealed.

## INTRODUCTION

*Escherichia coli* RNase E has a central role in controlling the cellular levels of all classes of RNA by mediating their processing or turnover or both (for recent reviews, see (1,2)). It is essential for cell viability and its contribution to RNA metabolism has been studied extensively using two temperature-sensitive mutations (3,4). These mutations cause amino acids substitutions (5) within an S1 RNA-binding domain that can close on a DNase I-like domain, which contains the catalytic residues, to form an elongated channel that accommodates unpaired (i.e. single-stranded) regions of RNA (6). Cleavage generates a downstream product with a 5′-monophosphorylated end (7) that can engage with a pocket located at one end of the RNA-binding channel (6). This 5′-‘sensing’ interaction probably ensures that any accessible sites further downstream are cleaved preferentially following an initial cleavage (8).

*Escherichia coli* and other bacteria contain RNA pyrophosphohydrolases (RppH in *E. coli*) that can convert the 5′ group of a primary transcript from a tri- to monophosphate (9). Moreover, the disruption of the *rppH* gene in *E. coli* results in the stabilization of many mRNA transcripts indicating that pyrophosphate removal is a significant route by which bacterial mRNA decay is initiated (10). However, only 20 to 25% of the detectable transcripts were stabilised indicating that an RppH-independent route(s) must exist to initiate the degradation of the majority of *E. coli* transcripts (11). Recently, it was shown *in vitro* that defined oligonucleotide substrates and sites within polycistronic tRNA precursors can be cleaved efficiently by RNase E in the absence of a 5′-monophosphorylated end. A proviso is that RNase E can contact an unpaired region(s) within the substrate in addition to the region in which cleavage occurs (12,13). Moreover, as intermediates of tRNA processing do not accumulate in cells that contain a 5′-sensor mutant as their only source of RNase E (14), it may be that no major aspect of tRNA maturation is critically dependent on 5′ monophosphate-dependent cleavage.

The ability of RNase E to cleave substrates efficiently in the absence of a 5′-monophosphorylated end reflects the tetrameric structure of the catalytic domain. This domain is formed by the dimerization of a dimeric unit that forms two symmetrical RNA-binding channels (6). Thus, the catalytic domain has the capacity to interact simultaneously with up to four unpaired regions. It is well established that the use of multiple regions of contact enhances the affinity and selectivity of macromolecular interactions (for review, see (15)). The catalytic domain of RNase E is located in its N-terminal half (NTH) (16), which is sufficient for cleavage *in vitro* at sites identified *in vivo* (13,14) and is conserved in many bacterial families and within plant plastids (17–19). The C-terminal half (CTH) contains ancillary RNA-binding domains and makes contacts that form the RNA degradosome and locate it to the inner surface of the cytoplasmic membrane (for reviews, see (1–2,20)). Two of the other components of the degradosome are polynucleotide phosphorylase, a 3′ to 5′ exonuclease (21), and RhlB, an RNA helicase (22). However, the CTH of *E. coli* RNase E is neither essential for cell growth (23,24) nor highly conserved (17,18) and likely represents a relatively recent evolutionary adaption that improves fitness by promoting the coupling of steps in RNA degradation (for review, see (25)).

Recent analyses of the molecular recognition that underlies RNA processing and degradation by RNase E have utilized mutations that substitute arginine 169 or threonine 170 within the pocket that engages 5′-monophosphorylated ends (12–13,26–28). Together these amino acids form a horseshoe of hydrogen bond donors that engage the monophosphate group (6). The substitution of the threonine at 170 with valine (T170V) reduces the efficiency of cleavage of 5′-monophosphorylated substrate by at least 10-fold, while the efficiency of cleavage of a 5′-hydroxylated equivalent remains low and largely unchanged (12). The use of the T170V mutant of NTH-RNase E was instrumental in confirming biochemically that the initial steps in the processing of at least some polycistronic tRNA precursors occurs via direct entry cleavage by RNase E (13). Here we used the same mutant in combination with controls and an RNA sequencing (RNA-seq) approach to investigate the repertoire of RNA cleavages mediated by direct entry. Our results indicated that direct entry by RNase E pervades in *E. coli* and may regulate gene expression in ways previously unexpected. New light is also shed on RNase G, a paralogue of RNase E.

## MATERIALS AND METHODS

### Strains

**Table:** 

Strain	Genotype (source)
BW25113	*rrnB3 ΔlacZ4787 hsdR514 Δ(araBAD)567 Δ(rhaBAD)568 rph-1* (29)
N3433	Hfr *lacZ43*(Fs), *λ*^−^*relA1, spoT1*, *thiE1* (30)
N3431	Same as N3433, but with *rne-3071* (ts) mutation (30)
MC1061	F^−^ Δ(*ara-leu*)7697 [*araD139*]_B/r_ Δ(*codB-lacI*)3 *galK16 galE15* λ^−^ e14^−^*mcrA0 relA1 rpsL150*(*str^R^*) *spoT1 mcrB1 hsdR2*(r^−^m^+^) (31)
GM11	Same as MC1061 with *rng::cat* mutation (32)

### Synthesis of RNA transcripts

Transcripts were synthesized *in vitro* using T7 RNA polymerase and polymerase chain reaction (PCR)-generated templates and purified as described previously (12,33). The concentration and integrity of RNA samples were determined using a NanoPhotometer^®^ P-300 (Geneflow) and agarose gel electrophoresis (33), respectively. The sequences of the primers used to generate templates are given in Supplementary Table S1.

### Annealing of complementary DNA oligonucleotides to *in vitro*-transcribed RNA

The sequences of oligonucleotide primers annealed to RNA transcripts to block access by RNase E are given in Table 1. The hybridization conditions and the RNase H-based assay used to confirm oligonucleotide binding were as described previously (13).

**Table 1.** List of oligonucleotides used in this study

Name	Primer sequence (5′ to 3′)
b1	TGGATAGTAAATTCCTGATCGTGC
b2	TGATACCAGTTGAGGATTAATTTCTCGACGGT
b3	TGATACCAGTTGAGGATTAATTTCTCGACGGTTTGAATATCACTGTCGAGGAATACGCCA
b4	GCAGTTTAAATTTTTTAATGATCTC
b5	GCCGGTAATTCTGCGGAATAC
b6	GGTGATATCCGGTCGATGG
b7	TCGGTTCAATGCGGGTGATT
b8	CGTTCAGCGCCGTAATCAAC
b9	TCTTTTAATTCGGTACGGTC
b10	CGGAAGCTTAAATCCCATTG
a1	AGATTGTTTCTTCGAAGG
a2	ACAAATTGGTTTTGAATTTGCCGAACATATTCGATACATTCAGAATT
a3	ACAAATTGGTTTTGAATTTGCCGAACATATTCGATAC
a4	TTGGGTGGTCTGTGCCTTACAGCACTTTCAAATTT
a5	GCGTCGCTGTGGATATTTTATTGAGAGAAGAATT
a6	GCGTCGCTGTGGATATTTTATTGAG

### Extraction of total RNA from *E. coli*

*Escherichia coli* strains used for the analysis of *in vivo* cleavages (30–32) were grown at 30°C, a widely used temperature that permits good growth of multiple ribonuclease mutants (34,35), while BW25113 (29), which was used as source of RNA for the analysis of *in vitro* cleavages, was grown at 37°C. All were incubated with shaking (200 rpm) in 250 ml Erlenmeyer flasks containing 50 ml of Luria Bertani (LB) broth (Sigma). At the midpoint in exponential growth (OD_600_ ∼0.6), a one-eighth volume of stop solution (95% [v/v] ethanol; 5% [v/v] phenol) was added to inhibit cell metabolism (33) and the cells were harvested by centrifugation. For the temperature-sensitive N3431 strain and its congenic wild-type (WT) N3433 partner, the cultures were shifted from 30 to 44°C for 45 min before the addition of stop solution. When necessary, cell pellets were stored frozen at −80°C. RNA was isolated as described previously (33) and enriched for mRNA using the MICROBExpress™ kit as described by the vendor (Ambion). To generate 5′-hydoxylated ends, the RNA was treated with tobacco acid pyrophosphatase (TAP; Epicentre^®^ Biotechnologies) and calf intestinal phosphatase (CIP; New England BioLabs) as described previously (13,33). To generate 5′-monophosphorylated ends, the RNA was incubated with polynucleotide kinase (PNK; New England BioLabs) as described previously (Kime et al., 2008) followed by TAP treatment (13). The 5′-phosphorylation status of the RNA was confirmed using Terminator™ 3′ to 5′ exonuclease (TEX; Epicentre^®^ Biotechnologies), an enzyme that is specific for 5′-monophosphorylated RNA when used in limiting amounts, as described previously (13).

### Purification of NTH-RNase E and discontinuous cleavage assays

Recombinant, N-terminally hexahistidine-tagged polypeptides corresponding to the NTH of RNase E (residues 1–529) with WT sequence or the T170V substitution were purified as described previously (12,33). Discontinuous cleavage assays were performed in a buffer containing 25 mM *bis*-Tris propane (pH 8.3), 100 mM NaCl, 15 mM MgCl_2_, 0.1% (v/v) Triton X-100, 1 mM dithiothreitol (DTT) and 32 U RNaseOUT™ ribonuclease inhibitor (Invitrogen). Reactions were started by combining enzyme (in buffer) with RNA substrate, both of which had been pre-incubated separately at 37°C for 20 min. Aliquots were taken at each time point and quenched by adding to an equal volume of 2x RNA loading dye; 95% (v/v) formamide, 0.025% (w/v) bromophenol blue, 0.025% (w/v) xylene cyanol and 0.025% (w/v) sodium dodecyl sulphate. The samples were analysed by denaturing polyacrylamide gel electrophoresis. For further details, see figure legends.

### Mapping of 5′-monophosphorylated ends by RNA-seq

Libraries of cDNA corresponding to 5′-monophosphorylated ends present before and after incubation with RNase E were constructed and sequenced as a service provided by vertis Biotechnologie AG (Germany). As described previously (36), the 5′-sequencing adaptor was ligated to transcripts prior to fragmentation, thereby allowing the 5′ ends of both long and short transcripts to be cloned. RNA was fragmented using a Bioruptor^®^ Next Gen UCD-300™ sonication system (Diagenode), then tailed at the 3′ end using poly(A) polymerase (New England BioLabs), copied into cDNA using M-MLV reverse transcriptase (RNase H minus, AffinityScript, Agilent) and an oligo-dT primer, amplified by PCR and fractioned by gel electrophoresis using an Agencourt AMPure XP kit (Beckman Coulter Genomics). Fragments of 200–500 bp were selected for sequencing, which was done using an Illumina HiSeq 2000 platform (single end, 50-bp read length). Reads were trimmed of 5′ adapter and poly(A) sequences and aligned against the genome of *E. coli* K-12 strain MG1655 (seq) (NCBI, accession number U00096.2).

## RESULTS

### Overview of approach

To assess the contribution of direct entry to RNA processing and degradation, a sample of *E. coli* RNA depleted of much of its 23S and 16S rRNA (i.e. enriched for mRNA) was incubated with NTH-RNase E (37) or the equivalent T170V 5′-sensor mutant (12). The dependency of cleavages on interaction with a 5′-monophosphorylated end was also investigated using samples that were predominantly either monophosphorylated or hydroxylated at the 5′ end. These were produced by treating samples enriched for mRNA with polynucleotide kinase (converts 5′-hydroxylated to 5′-monophosphorylated ends) followed by tobacco acid pyrophosphatase (TAP; 5′ triphosphorylated to monophosphorylated) or with TAP followed by calf intestinal phosphatase (CIP; 5′ monophosphorylated to hydroxylated), respectively. Positions of RNase E cleavage were then mapped using an RNA-seq approach specific for detecting 5′-monophosphorylated ends (for details, see ‘Materials and Methods’ section). The subtraction of 5′-monophosphorylated ends present before incubation identified those generated by RNase E *in vitro*. RNA-seq was used also to map the positions of sites that are highly dependent on RNase E *in vivo*. This was done by identifying 5′-monophosphorylated ends that were substantially depleted in an *rne*^ts^ strain of *E. coli* as a consequence of incubating at a non-permissive temperature. The baseline for the comparison was RNA isolated from a congenic WT strain that had been cultured under identical conditions. Our analysis then focussed on a selection of sites for which there was evidence of cleavage *in vivo*. This was done to exclude sites to which RNase E would not have access in growing *E. coli* as a consequence of, for example, ribosomes translating mRNA or proteins binding rRNA. Substrates containing a cleavage site(s) of interest were then characterized individually.

### Contribution of direct entry to RNA processing and degradation

**Figure 1.**
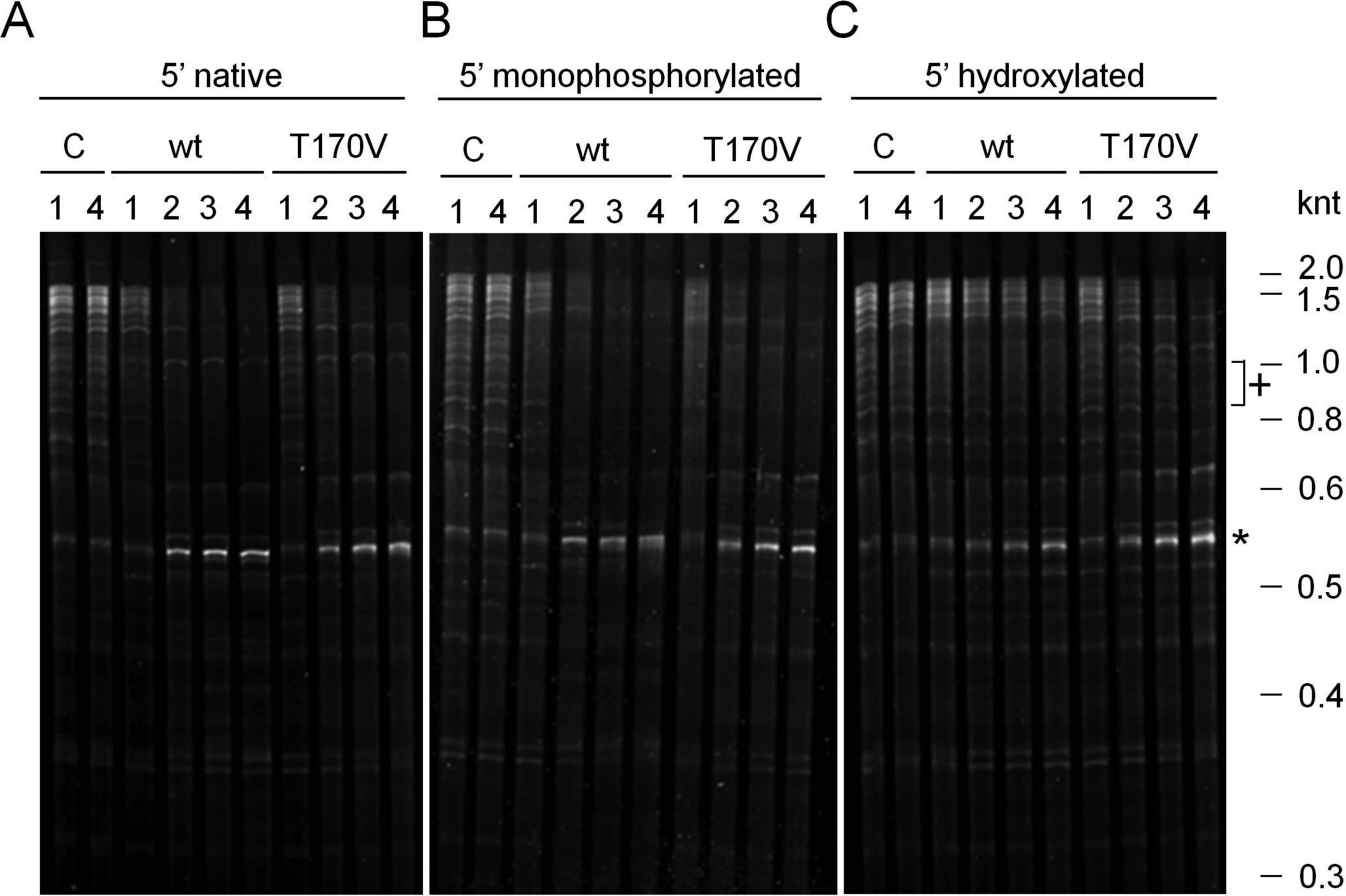
Assessment of the proportion of RNase E sites that are cleaved independent of interaction with a 5′-monophosphorylated end. NTH-RNase E with the wild-type (WT) sequence or T170V substitution were incubated with *Escherichia coli* RNA from strain BW25113 depleted of much of its 23S and 16S rRNA, i.e. enriched for mRNA. In (**A**) the 5′ ends were untreated, while in (**B**) and (**C**) they had been treated enzymatically to produce a monophosphate or hydroxyl group, respectively (see text for details). The reaction conditions, preparations of both WT NTH-RNase E and the T170V mutant, and the analysis of reactions using denaturing gel electrophoresis were as described recently (13). The concentration of RNase E (monomer) and enriched mRNA in each reaction was 300 nM and 30 ng/μl, respectively. Lanes 1–4 contain samples taken after 0, 10, 30 and 60 min. The RNA was stained using SYBR^®^ Gold Stain (Life Technologies). Labelling at the top of each panel identifies the 5′ status of the substrates and whether the substrates were incubated without enzyme (C, control), with WT NTH-RNase E (wt) or the 5′-sensor mutant (T170V). The positions of RNA size markers (not shown) are indicated on the right of the figure. An asterisk indicates an abundant product of direct-entry cleavage, while a cross indicates examples of RNA species that appear less susceptible to T170V when 5′ hydroxylated.

We found that a sample enriched for mRNA was cleaved extensively by both NTH-RNase E and T170V; moreover, there was no obvious difference in the pattern of cleavage (Figure 1, panel A). Thus, as a significant proportion of the native 5′ ends in *E. coli* RNA were expected to be monophosphorylated due to 5'-pyrophosphate removal ('decapping') or endonucleolytic cleavage, this provided the first indication that many, and possibly most, of the sites susceptible to RNase E in *E. coli* RNA can be cleaved independent of interaction with a 5′-monophosphorylated end. It should be noted that under the conditions used T170V was considerably slower than its WT counterpart at cleaving a 5′-monophosphorylated oligonucleotide substrate (Supplementary Figure S1). Results indistinguishable from those described above were observed when the RNA was treated to make the 5′ ends monophosphorylated, irrespective of their original status (Figure 1, panel B). The 5′-phosphorylation status of the bulk RNA was confirmed using Terminator™ exonuclease, a 5′ to 3′ exonuclease that is specific for 5′-monophosphorylated RNA when used in limiting amounts (Supplementary Figure S2, panel A). The 5′-hydroxylation status following treatment with TAP and CIP was also confirmed at the level of individual transcripts using RNA ligase-mediated RT-PCR (Supplementary Figure S2, panel B). In our experience, 5′ phosphates are removed more efficiently by treating with TAP followed by CIP than by treating with CIP only (unpublished observation). Obvious in all of the RNase E reactions described above was the accumulation of a distinct cleavage product of ∼550 nt. Moreover, as there was no corresponding decrease in the level of a longer species, this cleavage product (marked by an asterisk) was produced from multiple species. This cleavage product, which is identified below as a derivative of 16S rRNA, was also produced efficiently when the 5′ ends were dephosphorylated to make them hydroxylated (Figure 1, panel C). Thus, the formation of this product is a clear example of efficient cleavage by direct entry. However, dephosphorylation of 5′ ends did appear to result in some species (examples marked by a cross) becoming less susceptible to cleavage. These species are candidate substrates for 5′ monophosphate-dependent cleavage. Interestingly, NTH-RNase E appeared to cleave some of the longest RNA species in the 5′-hydroxylated RNA sample *less* efficiently than T170V (for possible explanation, see ‘Discussion’ section).

### Identification of sites cleaved by T170V *in vitro*

The next step in enumerating the contribution of direct entry to RNA processing and degradation was to identify the 5′-monophosphorylated products of incubating T170V with RNA that was dephosphorylated at the 5′ end. Libraries were prepared from aliquots of the 10 and 30 min timepoints and compared against an aliquot of the starting material. We prepared, sequenced and analysed libraries essentially as described previously by us (36), with the exception that additional rounds of PCR were required to amplify the cDNA to levels sufficient for the cloning step (for details, see ‘Materials and Methods’ section). This modification was required given that the vast majority of the 5′ ends in the starting material had been dephosphorylated and, as a result, could not be amplified. For each library, we mapped the genome positions of the 5′-monophosphorylated ends and obtained an estimate of the relative abundance of the corresponding fragments by counting the numbers of reads starting at each of these positions (36). The reads obtained before and after incubation with T170V were then compared using *M* (ratio)–*A* (intensity) scatterplots, where *M* = log_2_ (reads after/reads before incubation with enzyme), and *A* = (log_2_ [reads before] + log_2_ [reads after incubation])/2. Positions not associated with reads before and after incubation with enzyme were not included in the scatterplot. Where reads were obtained under only one condition, the read for the other was given a nominal value of 1 (the lowest limit of detection). For each timepoint, ∼600 000 ends were mapped.

**Figure 2.**
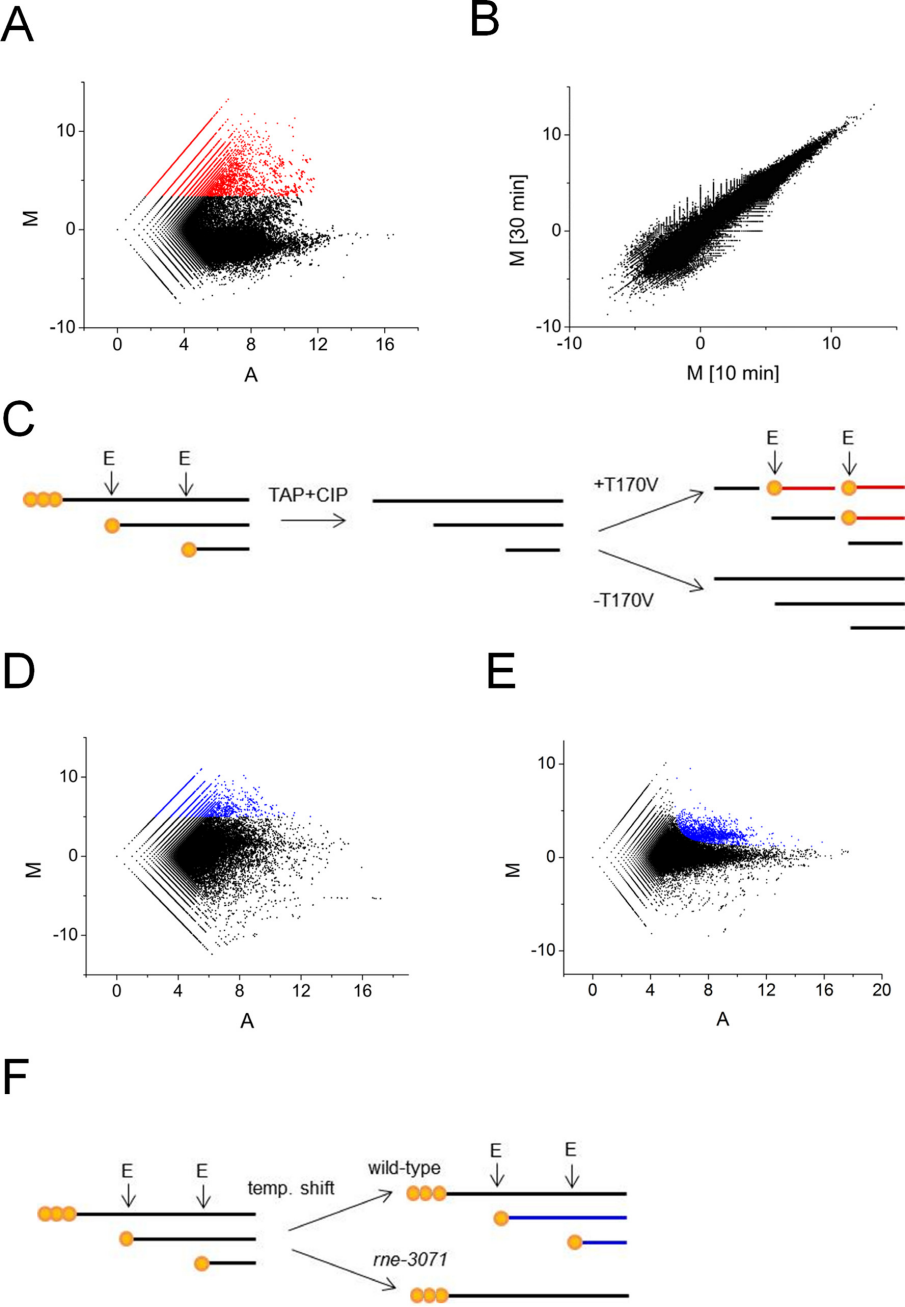
Scatterplot analyses of RNA-seq data. (**A**) shows a plot of *M* (ratio) and *A* (intensity) values corresponding to the reads obtained after and before incubation of 5′-hydroxylated RNA with T170V for 10 min. Each point corresponds to a unique 5′ end. The points coloured red have *M*-values ≥3.4. (**B**) is a plot of *M*-values obtained for panel A against *M*-values corresponding to reads obtained for the same reaction but after and before incubation for 30 min. (**C**) shows a schematic illustration of fragments generated in (A) with increased values of *M*. Primary transcripts, depicted with 5′-triphosphorylated ends (three orange circles) and the downstream products of cleavage, depicted with 5′-monophosphroylated ends (single orange circle), were isolated within total RNA isolated from *Escherichia coli*. Following mRNA enrichment, the RNA was treated with TAP and CIP so that all the ends were 5′ hydroxylated. Sites of direct entry *in vitro* were identified by sequencing the downstream products of cleavage (red fragments). The 5′-monophosphorylated ends of these fragments facilitated their cloning. For (A) and (B) enzyme monomer and enriched RNA from BW25113 concentrations were 300 nM and 30 ng/μl, respectively. (**D**) is a plot of *M* and *A*-values corresponding to reads obtained for an *rne-3071* strain and its congenic WT partner at 44°C, a non-permissive temperature. The points coloured blue have *M*-values ≥5. (**E**) as (D) except the values correspond to reads obtained for an *rng* disrupted strain and its WT partner. The points coloured blue correspond to candidate sites of RNase G cleavage *in vivo*. (**F**) shows a schematic illustration of fragments generated in (D) with increased values of *M*. Prior to the temperature shift, RNase E cleaves primary transcripts as part of their processing and degradation. This continues in the WT, but not the *rne-3071* strain at a non-permissive temperature. As a result, products of RNase E cleavage (blue fragments) can become depleted in the *rne-3071* strain.

Each of the scatterplots revealed a cone-shaped population of points that were distributed around an average *M*-value of −1.6 (data shown for 10 min timepoint; Figure 2, panel A). This population corresponds to 5′-monophosphorylated ends that were present in the starting material and were not generated by *in vitro* cleavage. The average *M-*value of this population was <0, as the generation of additional 5′-monophosphorylated ends reduced the level to which the starting material could be amplified. Above this population, there was a large ‘cloud’ of points of which ∼100 had *M*-values >10, ∼1000 had *M*-values >8, ∼13 500 had *M*-values >5, and ∼236 200 had *M*-values >2. When normalized against the average *M*-value of −1.6, the latter *M*-value of 2 corresponds to a fold increase of >10 after 10 min of incubation with T170V. A very similar pattern was observed for 5′ ends generated after 30 min of incubation with T170V, as illustrated using a scatterplot of the *M*-values obtained at the two timepoints (Figure 2, panel B). The coalescence of points along the diagonal of the *M-M* scatterplot (Spearman coefficient of 0.82 for *M*-values ≥3.4, *P*-value < 7 × 10^−6^) indicates that much of the cleavage was completed by 10 min and the RNA-seq approach provides a highly reproducible measure of the underlying enzymology. A schematic illustration of fragments that corresponds to 5′-end positions with increased values of *M* following incubation with T170V *in vitro* is provided (Figure 2, panel C).

### Mapping of sites dependent on RNase E *in vivo*

Next, sites that are highly dependent on RNase E *in vivo* were mapped by preparing libraries from enriched mRNA isolated from an *rne*^ts^ strain and its congenic WT partner at a non-permissive temperature. These libraries were then sequenced and analysed as described above, and the reads again compared using an *M*–*A* scatterplot (Figure 2, panel D). This time, however, *M* was log_2_(reads from WT/reads from *rne*^ts^ strain), and *A* was (log_2_[reads from WT] + log_2_[reads from *rne*^ts^ strain])/2. This revealed a wide scatter of points with *M*-values considerably below as well as above the average. The wide scatter was expected, as the inactivation of RNase E is known to stabilize degradation and processing intermediates as well as block the generation of others. In contrast, a scatterplot analysis of libraries prepared from enriched mRNA from an *rng* disruption strain and its congenic WT partner (Figure 2, panel E) reveal a cone of values centred on an *M*-value of 0 with a relatively tight cloud of points with higher *M*-values. This pattern is entirely consistent with RNase G having a much more restricted role in RNA metabolism (38,39). A schematic illustration of fragments that corresponds to 5′-end positions with increased values of *M* following inactivation of RNase E *in vivo* is provided (Figure 2, panel F).

**Figure 3.**
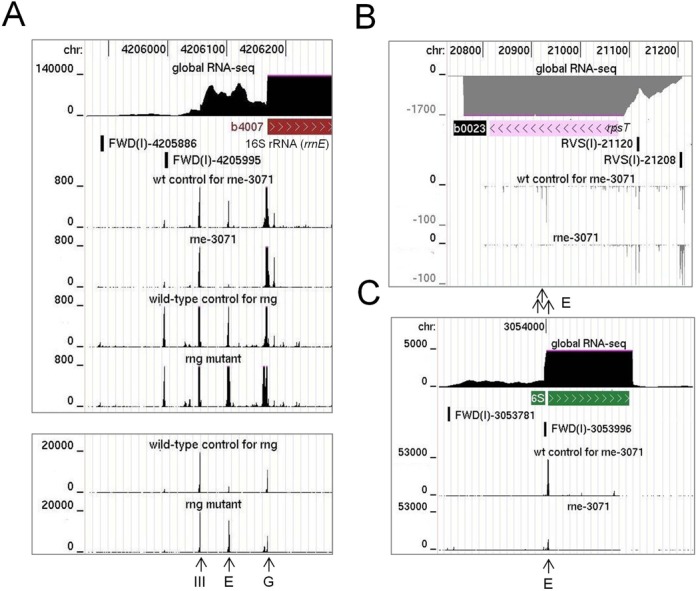
RNA-seq profiles at well-documented sites of RNase E cleavage. (**A**), (**B**) and (**C**) show the profiles for sites at the 5′ end of 16S rRNA, within the coding region of *rpsT* mRNA and at the 5′ end of 6S RNA, respectively. In each panel, the top three tracks show the positions of the corresponding gene with the transcriptome (global RNA-seq) above and the transcription start site mapping data below for a WT strain of Escherichia *coli* grown under similar conditions (Romero *et al.*, submitted). The tracks below show the reads obtained at each position in a mutant and its congenic WT partner. The strains are identified by labels above each track. Numbers on the left indicate the scale of the sequencing reads, while numbers at the top indicate the genome position. Data for the disruption of RNase G is shown using two different scales in (A). The panels are modified screenshots from the UCSC Microbial Genome Browser (40). Vertical arrows at the bottom of each panel identify sites of cleavage by RNase III, E and G.

Viewing of the RNA-seq data for the inactivation of RNase E *in vivo* using a genome browser (40) confirmed that substantial reductions in sequence reads were obtained at the positions of well-documented sites of RNase E cleavage. This included the RNase E sites mapped (i) 66 nt upstream of the mature 5′ end of 16S rRNA (41,42), (ii) within the coding region of *rpsT* mRNA (43), (iii) at the 5′ and 3′ end of pre-5S rRNA (44), (iv) within the tRNA precursor of *argX*-*hisR*-*leuT*-*proM* (13,45), (v) within the intergenic region of *pyrG-eno* mRNA (39), (vi) just within the 3′ end of the coding region of *epd* mRNA (46), (vii) at the 3′ end of the coding region of *dnaG* mRNA (47), (viii) at the 5′ end of 6S RNA (48) and (ix) at the 3′ end of tmRNA (49). Cleavage detected at the 5′ end of 16S rRNA is shown as one of three examples; RNA-seq data for the inactivation of RNase G has been included (Figure 3, panel A). The heights of the peaks represent the abundance of 5′ ends detected at each position by RNA-seq. Cleavage at the RNase E site (position −66; relative to 5′ end of mature 16S rRNA) follows cleavage by RNase III (position −155) and mediates efficient cleavage by RNase G at the 5′ end of 16S rRNA (41,42). Consistent with this path, inactivation of RNase E and G results in the number of reads at position −66 decreasing and increasing, respectively. Interestingly, cleavage by an unknown nuclease was detected at position −5 following inactivation of RNase G. RNase E cleavage detected within the coding region of *rpsT* mRNA and at the 5′ end of 6S rRNA are also shown as examples (Figure 3, panel B and C). In all cases, the reads associated with RNase E sites are reduced significantly following inactivation of the enzyme. It should be noted that reads associated with sites thought to be cleaved exclusively by RNase E can still be obtained following the temperature shift, as species produced prior will not necessarily have been degraded to completion. The number of RNase E-dependent sites with *M*-values ≥5 was 6997. Of these, 1852 were also cleaved by T170V *in vitro* (*M*-values ≥3.4, which is ≥5 above the baseline of −1.6; see Figure 2).

### Sites dependent on RNase G *in vivo*

**Figure 4.**
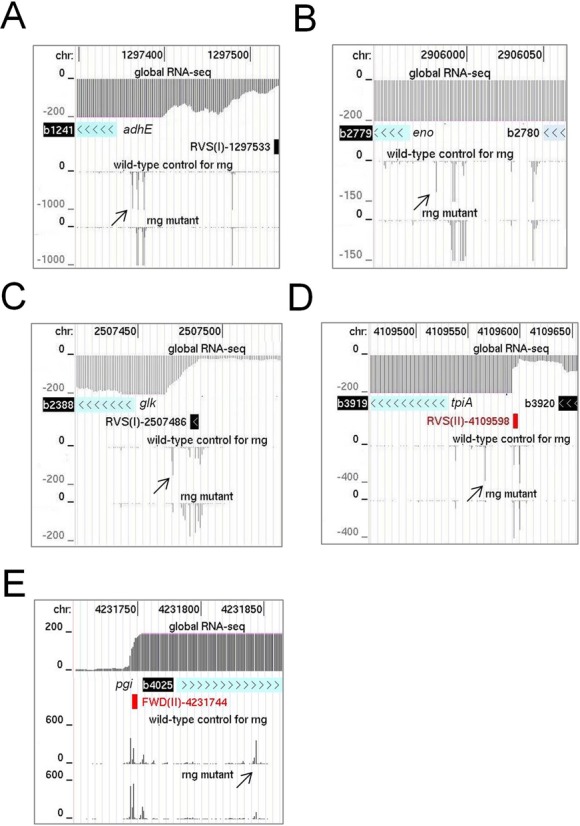
Sites of *rng*-dependent cleavage within mRNA. (**A**) and (**B**) show the RNA-seq profiles for sites at the 5′ end of *adhE* and *eno* mRNA. (**C**), (**D**) and (**E**) show sites within *glk*, *tpiA and pgi* mRNA, respectively. For each mRNA, the top three tracks show the positions of the corresponding gene with the transcriptome (global RNA-seq) above and the transcription start site mapping data below for a WT strain of Escherichia *coli* grown under similar conditions (Romero *et al.*, submitted). The tracks below show the reads obtained at each position in the *rng* mutant and its congenic WT partner. Diagonal arrows identify RNase G sites described in the main text. Numbering and labelling as Figure 3.

Within our RNA-seq data for the RNase G disruption strain and its congenic WT, which was included primarily as a reference for the inactivation of RNase E, we identified *rng*-dependent sites within *adhE* and *eno* mRNA. Both of these transcripts are stabilized by disruption of RNase G (28), resulting in increased rounds of translation (28,32,50). For both examples, a major site of RNase G-dependent cleavage was evident within the 5′ leader of the mRNA (Figure 4, panels A and B). The site in *adhE* mRNA had been mapped earlier, but not assigned initially to RNase G (51). Recently, a detailed functional analysis confirmed that this is indeed a site of RNase G cleavage (52). Cleavage at both the major site in *eno* and the one in *adhE* mRNA shortens the 5′ leader to 18 nt and may reduce translation by diminishing the efficiency of initiation. It may also stimulate cleavage further downstream; reduced levels of endonucleolytic cleavage at secondary sites were detected upon disruption of RNase G (data not shown). Concomitant with the disruption of RNase G cleavage was the accumulation of species produced by tight clusters of endonucleolytic cleavage upstream, which is evident at the scale shown by the broadening of the corresponding peaks. We also identified similar RNase G-dependent sites upstream of the coding region of the mRNA of *glk*, *tpiA* and *pgi* (Figure 4, panels C, D and E), three of four mRNAs that along with *eno* and *adhE* mRNA accumulated >2-fold upon disruption of RNase G (38). Cleavage at the 5′ end of the other mRNA that accumulated, *clpB*, was obscured by an overlapping small RNA encoded on the *same* strand (data not shown).

### Confirmation of direct entry cleavage using defined transcripts

To confirm that RNase E sites identified by our combined RNA-seq approach are cleaved efficiently independent of interaction with a 5′-monophosphorylated end, we generated 5′-triphosphorylated fragments containing these sites by *in vitro* transcription and incubated them with T170V. Sites with the highest *M*-values following incubation of the enriched mRNA with T170V *in vitro* were selected from those with a minimum *M*-value of 5.0 following inactivation of RNase E *in vivo*. A list of 100 sites with the highest *M*-values obtained *in vitro* is provided (Supplementary Table S2). Interestingly, this list includes sites within the mRNA of RNase E and RNase III suggesting possible roles for direct entry in the auto- and cross-regulation of ribonuclease activity, respectively. The list also includes (i) a site at position +390 (relative to the start codon) within *ompA* mRNA, which is well documented as having a 5′ stem-loop that blocks 5′-end-dependent cleavage (53), (ii) sites within several precursors of tRNAs (e.g. *hisR*, *proM*, *glyX*), which is consistent with the results of a recent publication (13), (iii) a site within 23S rRNA that maps to the evolutionarily conserved helix/loop 70 in the active centre of the ribosome (for review, see (54)) and (iv) a site at position +83 (relative to the transcriptional start site) within the Hfq-binding region of FnrS regulatory RNA, which reprogrammes metabolism in response to anaerobiosis (55,56). The cleavage that produces the prominent ‘0.55 knt’ species in our initial cleavage assays (Figure 1) corresponds to the +559 site in 16S rRNA, which had *M*-values for the *in vitro* and *in vivo* analyses of 7.0 and 5.8, respectively.

**Figure 5.**
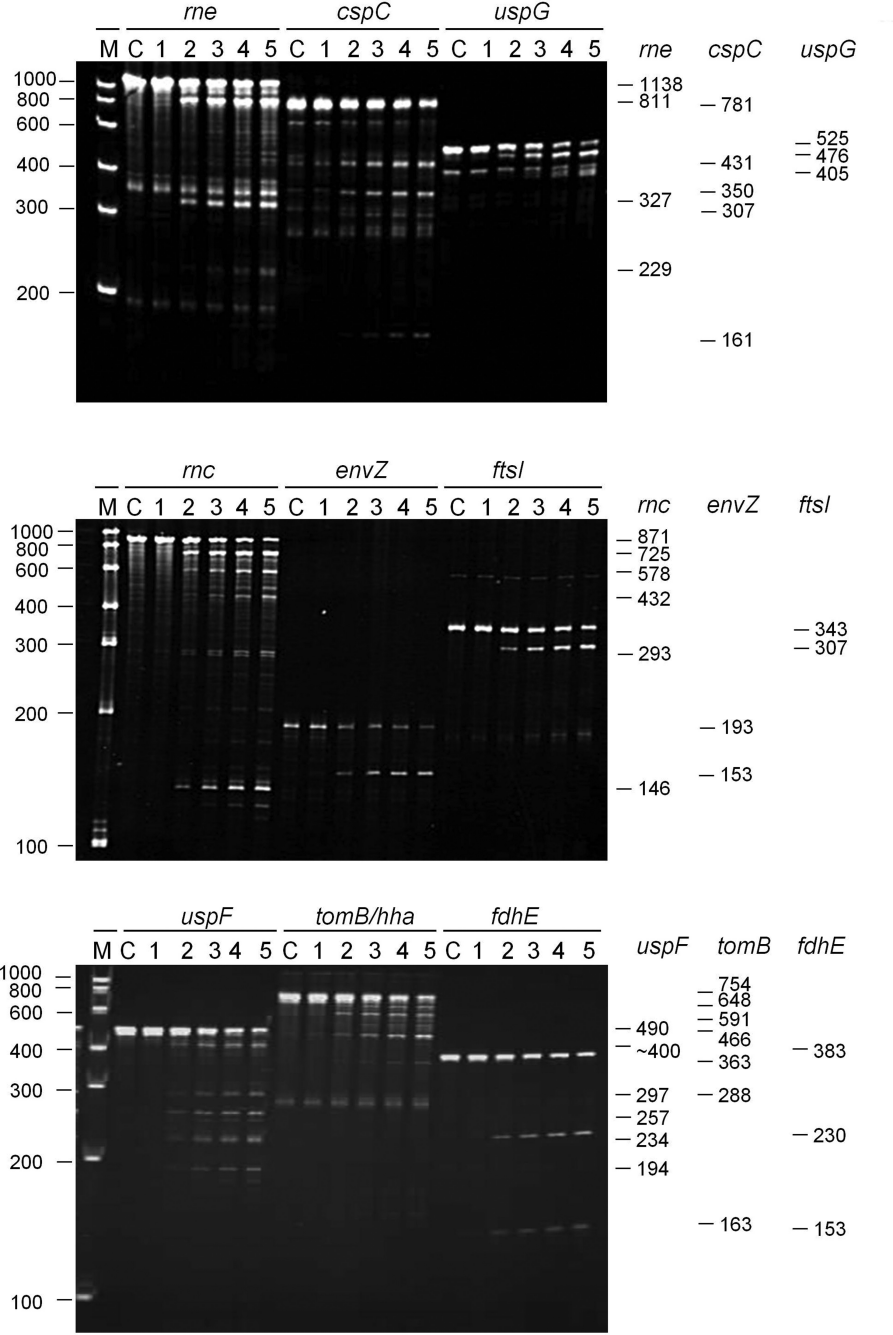
Efficient cleavage of 5′-triphosphorylated mRNA fragments by direct entry. The genes to which each of the 5′-triphosphorylated RNA fragments correspond are indicated at the top of each panel. The 5′ and 3′ boundaries of each of the 5′-triphosphorylated mRNA fragments, numbered relative to the start codon of the corresponding gene, are as follows: *rne*, −361 to +774; *cspC*, −202 to +576; *uspG*, -38 to +484; *rnc*, −160 to +708; *envZ*, +165 to +355; *ftsI*, +1,420 to +1,767; *uspF*, −22 to +465; *tomB/hha*, −89 to +662; and *fdhE*, −135 to +245. The sites in the region encompassing the 3′ end of *tomB* and 5′ end of *hha* are numbered relative to the start of *tomB*. The 5′-triphosphorylated transcripts were generated by *in vitro* transcription using conditions described previously (13,33). Each transcript has an additional GGG at the 5′ end generated during *in vitro* transcription by T7 polymerase. The conditions for the cleavage assays, the preparation of T170V, and the analysis of reaction products by denaturing gel electrophoresis were also as described previously (13,33). The enzyme monomer and initial substrate concentrations at the start of each reaction were 20 and ∼180 nM, respectively. The RNA was stained using ethidium bromide. Lanes 1–5 contain samples taken 0, 5, 15, 30 and 60 min, respectively, after mixing substrate and enzyme. Lane C contains substrate incubated without enzyme for 60 min. The sizes (nt) of RNA markers (Thermo Scientific RiboRuler Low Range) are indicated on the left of the panel. The sizes (nt) of each of the substrates and the major products are provided on the right. The sequences of the oligonucleotides used to generate the templates for *in vitro* transcription are provided (see ‘Materials and Methods’ section).

The incubation of 5′-triphosphorylated fragments with T170V identified cleavages that were efficient, relative to those in *cspA* mRNA and the *argX-hisR-leuT-proM* tRNA precursor (Supplementary Figure S3), in *rne*, *cspC*, *uspG*, *rnc*, *envZ*, *ftsI*, *uspF*, *tomB/hha* and *fdhE* mRNA (Figure 5). The efficiencies of cleavage can be estimated from the half-lives of the substrates, which reflected measurements of the initial rate in all cases (data not shown). Moreover, the results of truncating mRNA transcripts or blocking sites using complementary oligonucleotides (data not shown) are consistent with all of the fragments being cleaved at sites identified by the RNA-seq analyses (Figure 2). However, the major sites of cleavage observed using defined substrates were not always the ones with the highest *M*-values for T170V cleavage of enriched mRNA. This was not unexpected and, as discussed further below, probably reflects the fact that the 5′ and 3′ boundaries of the defined substrates were almost certainly different from those of the substrates in *E. coli*. Regardless of this difference, these results provide further evidence that direct entry, i.e. efficient cleavage by RNase E in the absence of binding to a 5′-monophosphorylated end, extends well beyond the maturation of tRNA.

### A role for adjacent unpaired regions in mediating direct entry appears wide spread

**Figure 6.**
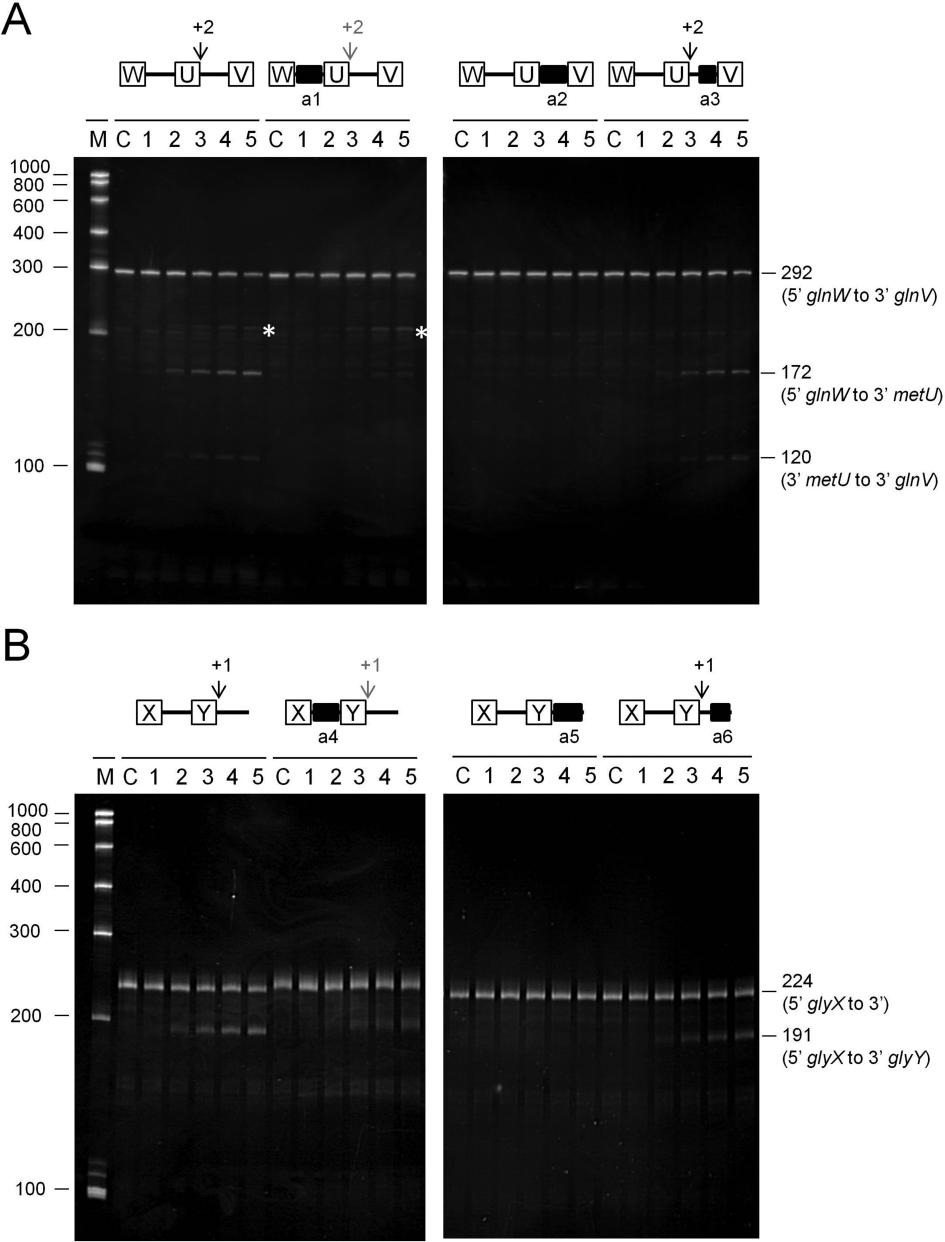
Analysis of direct entry cleavage in *metT* and *glyV* tRNA precursors. The cleavage assays, analysis of products and labelling are as Figure 5. (**A**) and (**B**) show the analysis of the *glnW-metU-glnV* and *glyX-glyY* tRNA precursors, respectively. A schematic diagram showing the positions of the sites of direct entry-cleavage (vertical arrow) and binding of a complementary oligonucleotide (closed black box) is provided for each transcript. White asterisks in (A) identify the production of a 209-nt species that is described in the main text.

Previously, we have shown that access to specific unpaired regions is required for direct-entry cleavage at adjacent, but non-contiguous sites in the *argX-hisR-leuT-proM* tRNA precursor. This finding is not specific to this precursor (Figure 6). A 292-nt fragment of the *metT*-*leuW-glnU*-*glnW*-*metU*-*glnV-glnX* tRNA precursor is cleaved at a site 2 nt downstream of *metU*. This cleavage can be blocked by annealing an oligonucleotide (labelled a1; see Table 1) to the intergenic region upstream between *glnW* and *metU*, as evidenced by a substantial reduction in the amount of the 172 and 120 nt products (hereafter all products are ordered upstream and downstream, respectively) (panel A). Annealing of an oligonucleotide (labelled a2) to the *metU-glnV* intergenic region confirmed the location of the cleavage. For the site 2 nt downstream of *metU*, the *M*-values for the *in vitro* and *in vivo* RNA-seq analyses (Figure 2) were 8.8 and 1.4, respectively. Hereafter the equivalent values for other sites are provided in parentheses. In the background, the production of a species of 209 nt (marked by a white asterisks) continued to be detected after the annealing of the a1 oligonucleotide. This corresponds to cleavage at a site 39 nt downstream of *metU* (*M*-values of 6.0 and 0.7, respectively). The location of this second site was confirmed by annealing an oligonucleotide (labelled a3) to just the 3′ side of the intergenic region between *metU-glnV*, but the requirements for cleavage at this site were not investigated further.

Analysis of a 224-nt fragment of the *glyV-glyX-glyY* precursor revealed efficient cleavage at a site 1 nt downstream of *glyY* (*M*-values of 7.4 and 2.6, respectively). Similar to the findings described above, this was reduced substantially by annealing an oligonucleotide (labelled a4) to the intergenic region upstream between *glyX* and *glyY*, as evidenced by the reduction in the amount of the 191-nt upstream product (panel B). The 33-nt downstream product was too small to be detected. The finding that cleavage was blocked by the annealing of an oligonucleotide (labelled a5) to the entire region 3′ to *glyY*, but not the annealing of an oligonucleotide to only the 3′ half of this region (labelled a6), confirmed the location of the corresponding site. Thus, contrary to our initial interpretation (13), the 3′ side of *glyY* is the location of one of the major sites of RNase E cleavage within the *glyV-glyX-glyY* precursor *in vitro*.

**Figure 7.**
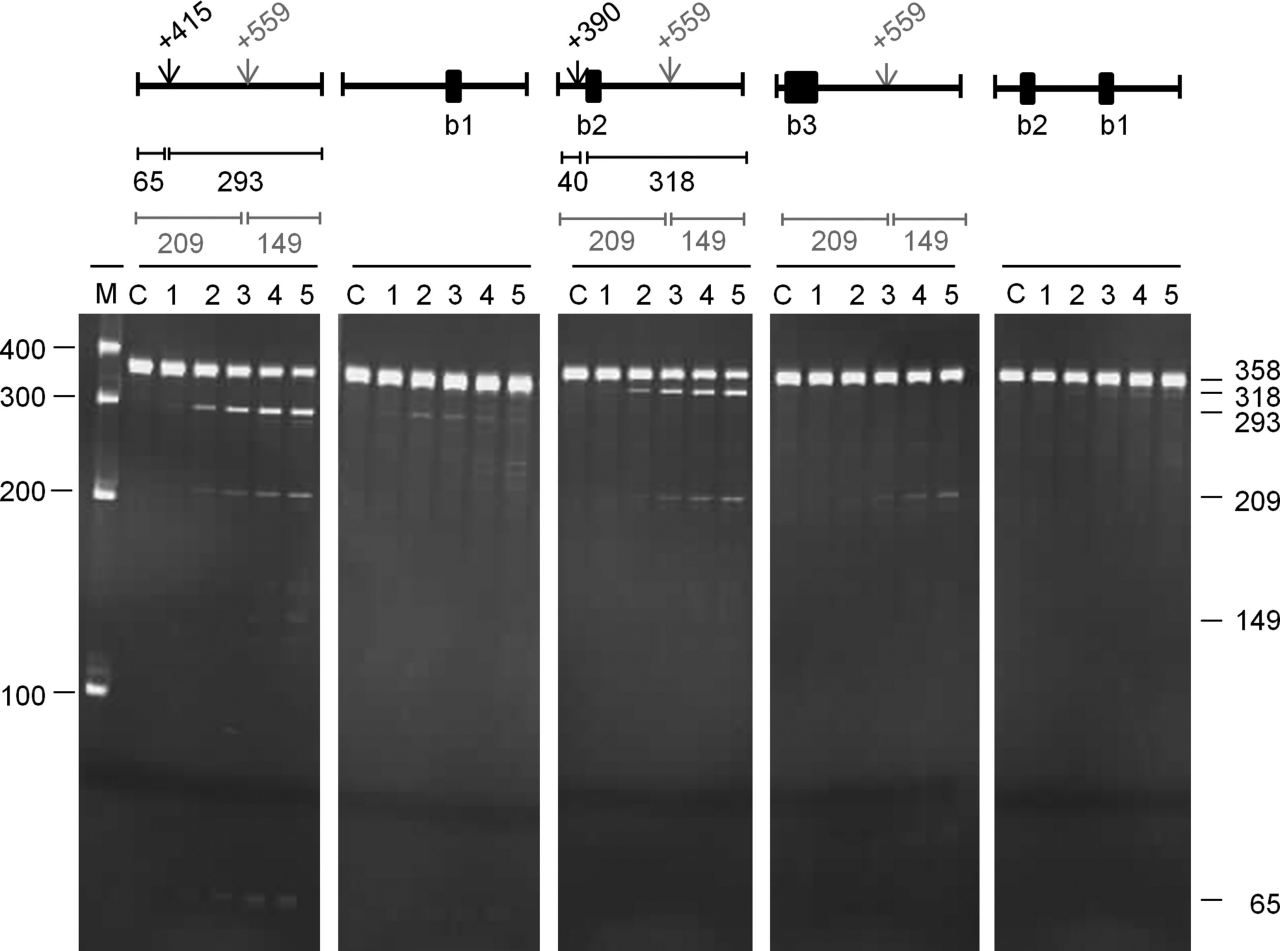
Analysis of direct entry cleavage in *rnc* mRNA. The substrate was a 358 nt transcript corresponding to +354 to +708 relative to the start codon. The cleavage assays, analysis of products and labelling are as Figure 5. A schematic diagram showing the positions of the sites of direct entry-cleavage (vertical arrow) and binding of a complementary oligonucleotide (closed black box) is provided for each transcript. The sizes of the products generated as a result of cleavage are also included.

We also investigated the requirements for direct entry cleavage of mRNA using, as examples, *rnc*, *uspF* and *rne*. In the case of *rnc* (Figure 7), a 358-nt fragment corresponding to the 3′ half of the transcript was cleaved primarily at position +415 relative to the start codon (*M*-values of 4.3 and 5.6, respectively), as evidenced by products of 293 and 65 nt. It was also cleaved significantly at position +559 (*M*-values of 9.2 and 5.5, respectively) as evidenced by products of 209 and 149 nt. Annealing an oligonucleotide (labelled b1) to the unpaired region encompassing +559 blocked cleavage at +415 (as well as +559). In contrast, annealing an oligonucleotide (b2) to the unpaired region encompassing +415 shifted the position of the cleavage ∼25 nt upstream, without affecting the efficiency of cleavage at +559. The annealing of an oligonucleotide (b3) that extended to the shifted site blocked the corresponding cleavage and, as with the shorter oligonucleotide (b2), did not affect the efficiency of cleavage at +559. Cleavage at the shifted site was also dependent on access to the region encompassing position +559, as evidenced by the complete blocking of cleavage when both the b1 and b2 oligonucleotides were annealed at the same time. A shift in the position of RNase E cleavage also occurred when an oligonucleotide was used to block a region encompassing the site in *fdhE* mRNA listed in Supplementary Table S2 (data not shown) and in *uspF* (see below). The results described above suggest that, as reported originally for tRNA precursors (13), an unpaired region of RNA recognised by RNase E (i.e. encompassing position +559) can be cleaved or facilitate cleavage in others (i.e. encompassing position +415). The requirements for cleavage at +559 have not yet been identified. Further analysis of the *rnc* transcript has revealed however that cleavage at +415 can also be blocked by the annealing of an oligonucleotide complementary to the region +658 to +681 (data not shown). This may represent the first example of direct-entry cleavage that requires simultaneous access to two additional unpaired regions.

**Figure 8.**
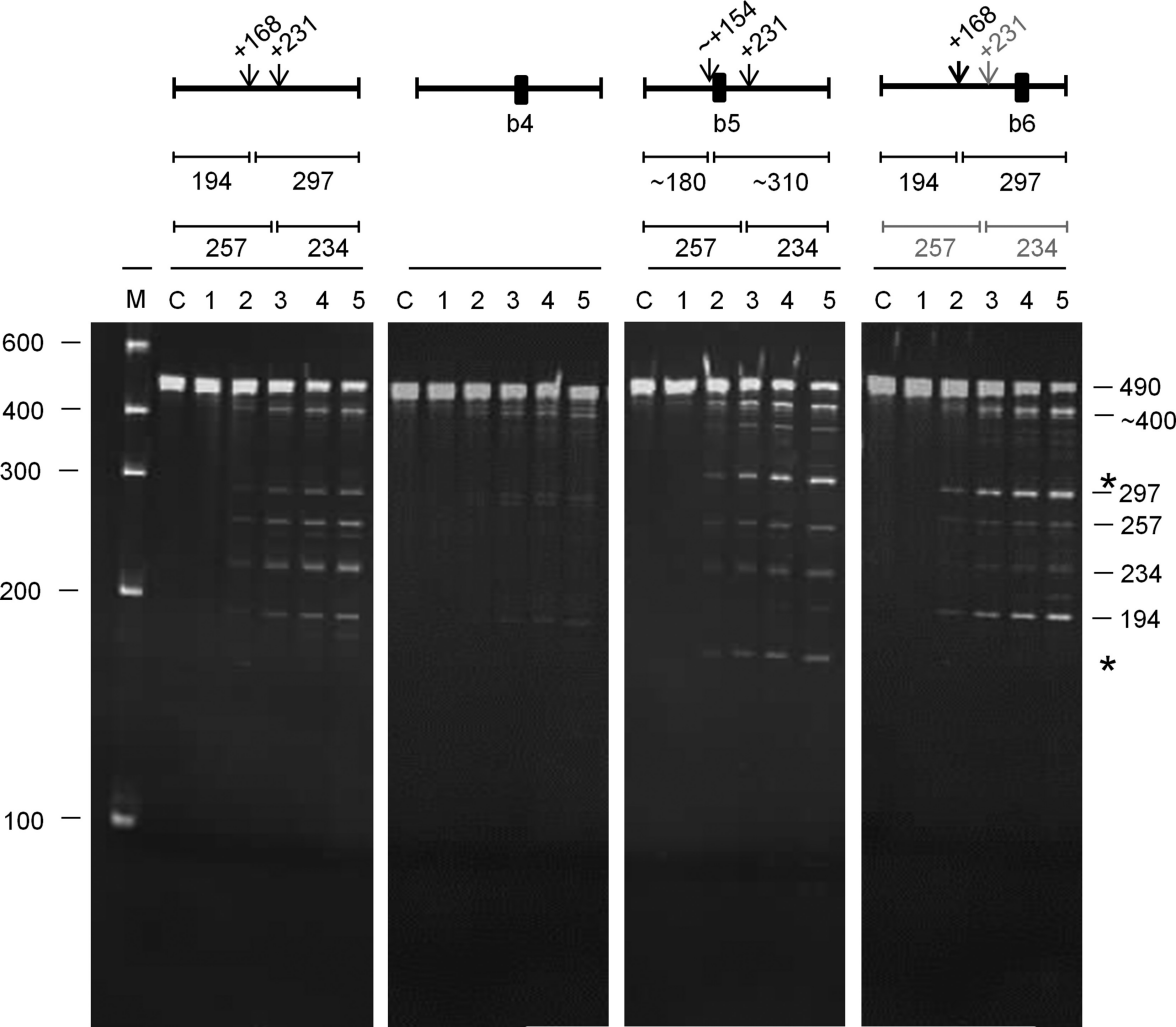
Analysis of direct entry cleavage in *uspF* mRNA. The substrate was a 490-nt transcript corresponding to −22 to +465 relative to the start codon. The cleavage assays, analysis of products and labelling are as Figure 7. The asterisks to the right of the panel indicate the positions of the products of a shifted cleavage (see main text).

For *uspF* (Figure 8), a 490-nt fragment corresponding to positions −22 to +465 relative to the start codon was cleaved at position +231 (*M*-values of 9.7 and 3.9, respectively), as evidenced by products of 257 and 234 nt. It was also cleaved efficiently at position +168 (*M*-values of 5.2 and 2.2, respectively), as evidenced by products of 194 and 297 nt. The abundance of the 297-nt product was less than the 194-nt product suggesting that the 297-product was also a substrate for cleavage at position +231. Less efficient, although still readily detectable, cleavage at an unmapped site produced a species of ∼400 nt. Annealing an oligonucleotide (b4) to the unpaired region encompassing position +231 reduced cleavage at position +168 (as well as blocking cleavage +231), as evidenced by the significant reduction in the accumulation of the 194 and 297 nt products. In contrast, annealing an oligonucleotide (b5) to the region encompassing position +168 had little, if any, effect on cleavage at +231; products of 257 and 234 nt were still detected readily. It did, however, shift cleavage from +168 to a site farther upstream as evidenced by products of ∼310 and ∼180 nt (marked with an asterisk). This shift in cleavage, as well as the finding that the recognition requirements for cleavage at +168 and +231 are not equivalent, resembles the situation described above for *rnc* mRNA (Figure 7). To identify a region required for efficient cleavage at +231, we reasoned that it could involve a site that is in some context cleaved by RNase E. An obvious candidate was the unpaired region encompassing position +353, which was associated with a high *M*-value for the *in vitro* RNA-seq analysis (Supplementary Table S2). The annealing of an oligonucleotide (b6) to this region reduced, but did not block, cleavage at position +231. As a consequence, cleavage at +168 was slightly enhanced. Incomplete blocking of cleavage at +231, did not appear to be associated with incomplete annealing of the oligonucleotide (data not shown) suggesting the existence of a second interaction that can mediate cleavage at the +231 site.

**Figure 9.**
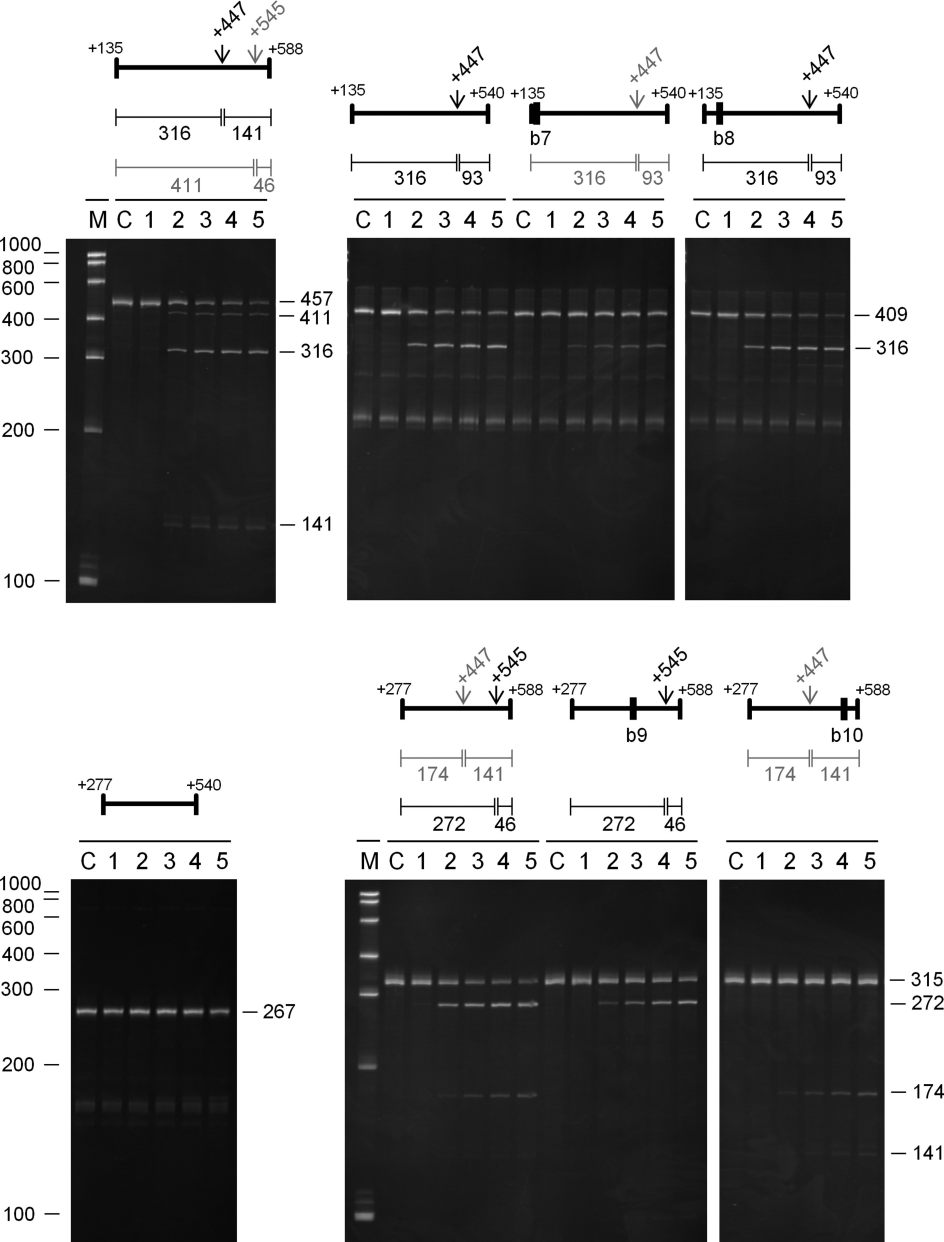
Analysis of direct entry cleavage in *rne* mRNA. The start and end positions (relative to the start codon) for each substrate are labelled above each image. Additional labelling as Figure 7.

For *rne* (Figure 9), a 457-nt fragment corresponding to a region within the 5′ half of the coding region was cleaved at two sites. The major site was at +447 (*M*-values of 8.5 and 4.7, respectively) as evidenced by products of 316 and 141 nt. Cleavage at +545 (*M*-values of 8.1 and 5.0, respectively) was also detected, as evidenced by the upstream product of 411 nt, but to a lesser extent. Efficient cleavage at +447 was unaffected by trimming 48 nt from the 3′ end to remove the +545 site (see 409-nt substrate). The 316-nt product common to this and the previous reaction accumulated at the same rate. Thus, as described above for sites in other substrates, the requirements for cleavage at +447 and +545 are not equivalent. Maximum cleavage at +447 appears to require access to an unpaired region between +135 and +152, as demonstrated by the slower rate of accumulation of the 316-nt species upon the annealing of a complementary oligonucleotide (b7). Annealing of an oligonucleotide (b8) to an adjacent region (+172 to +191) had no effect. The residual cleavage detected upon blocking of the region between +135 and +152 suggests the existence of second interaction that can produce cleavage at the +447 site. This involves at least one region between +135 and +277, as removing this region blocked cleavage at the +447 position (cf. cleavage of 409- and 267-nt substrates). Interestingly, cleavage at the +447 could be restored by adding back the 48-nt region between +541 and +588, as evidenced by the accumulation of the upstream product of 174 nt (cf. cleavage of 315- and 267-nt substrates). The corresponding downstream product is cleaved at +545 to produce smaller species (data not shown). Thus, it appears that cleavage at +447 can be supported by elements on its 5′ or 3′ side (cf. cleavage of 267-nt substrate with 409- and 315-nt substrates, respectively). The restoration of cleavage at the +447 site in the 315-nt substrate does not require access to the +545 site itself as the annealing of an oligonucleotide (b10) to this region had no effect on the rate of accumulation of the 174-nt product. The requirements for cleavage at +545 were not investigated beyond showing that access to the region encompassing +447 is required for maximum cleavage, as evidenced by the slower rate of accumulation of the 272-nt product when an oligonucleotide was annealed to the +447 site (b9). A requirement for the unpaired region encompassing the +447 site explains why cleavage at +545 was enhanced when cleavage at +447 was diminished by the removal of the 142-nt region at the 5′ end of the original fragment (cf. cleavage of 457- and 315-nt substrates).

## DISCUSSION

### Pervasive direct entry cleavage

We show here using an approach that incorporated RNA seq (57,58) that one of the hallmark properties of RNase E, i.e. its ability to interact with 5′-monophosphorylated ends, is unnecessary for efficient cleavage at a plethora of sites within the *E. coli* transcriptome. A preparation of RNase E with the T170V substitution, which disables interaction with a 5′-monophosphorylated end (12), was able when incubated with 5′-hydroxylated *E. coli* RNA (Figure 1) to reconstitute cleavage at many sites of RNase E-dependent cleavage *in vivo* (Figure 2). These sites map within mRNA, tRNA, rRNA and sRNA transcripts and can be viewed using our RNA-seq data, which has been deposited in the NCBI GEO repository (59). The cleavage of a selection of defined 5′-triphosphorylated transcripts, which were synthesized by *in vitro* transcription, confirmed that interaction with a 5′-monophosphorylated end was unnecessary (Figure 5). Thus, contrary to earlier expectations (60), the recognition of substrates by direct entry (24,46,61–63) pervades in RNA metabolism in *E. coli* and probably many other bacterial species that contain homologues of RNase E (18,19).

### Direct entry: a flexible mode of RNase E cleavage

**Figure 10.**
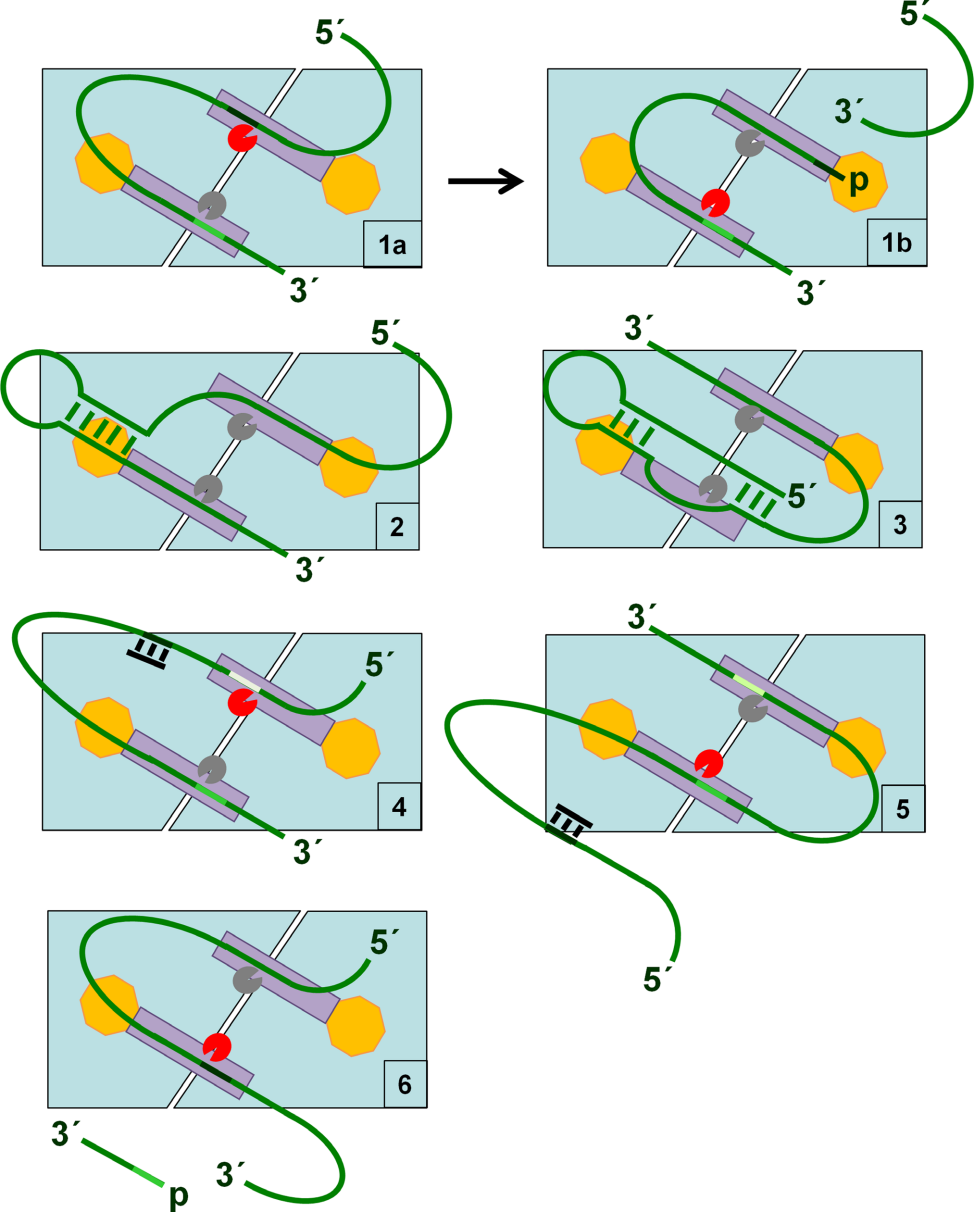
Direct entry: a flexible mode of RNase E cleavage. The principal dimer of RNase E is shown schematically (pale blue). It can interact simultaneously with RNA (green strand) via two unpaired regions (handholds) using its two equivalent RNA-binding channels (dark grey rectangles) (panel 1a). Whilst grasping two unpaired regions, one can be cleaved. The active sites are indicated by pacman symbols, which are coloured red when preferential cleavage of the corresponding unpaired regions is described. Cleavage of the 5′ most site (panel 1a) produces a 5′-monophosphorylated end that, following repositioning within the channel, provides a foothold for the 5′ sensor domain (yellow octagon), thereby extending the overall interaction to facilitate cleavage within the second handhold (panel 1b). As we have shown, unpaired regions that provide handholds can be separated by folded structures (panel 2). We also envisage that one or more of the handholds can itself be part of a folded structure (panel 3). There also appears to be flexibility in the selection of unpaired regions for handholds. We have described in this study examples where the annealing of a complementary oligonucleotide (black bar) to an unpaired region in which cleavage would otherwise have occurred does not block cleavage *per se*, but results in it occurring within an adjacent unpaired site (panel 4). We have also described examples where it appears that cleavage within a particular unpaired region can be facilitated by any one of several additional handholds. Thus, blocking (as illustrated) or removing a handhold does not necessarily prevent cleavage (panel 5). Should cleavage occur first within the most 3′ of two unpaired regions used for binding, it is envisaged that cleavage of the most 5′ region will be facilitated in a subsequent step by RNase E using another unpaired region as a handhold (panel 6) or perhaps a 5′-monophosphorylated end as a foothold (not shown). The unpaired region that serves as a handhold (panel 6) although shown to be located upstream of the region that is cleaved could be located downstream. The actual sequences of unpaired regions used in binding are likely to determine, at least in part, which is cleaved preferentially (65,66).

We provide evidence that, as found recently for the *argX-hisR-leuT-proM* tRNA precursor (13), direct entry cleavage at sites within *rne*, *rnc* and *uspF* mRNA as well as the *metT-leuW-glnU-W-metU-glnV-X* and *glyV-X-Y* tRNA precursors requires access to unpaired regions in addition to those that are cleaved (Figure 6). As discussed previously, the simultaneous binding of RNase E to two or perhaps more unpaired regions will increase the affinity of the overall interaction (12,13). Moreover, evidence is presented here that RNase E possesses flexibility with regard to the binding of unpaired regions. The annealing of oligonucleotide complementary to the unpaired regions encompassing the +415 position in *rnc* mRNA and the +168 site in *uspF* mRNA did not block, but rather shifted cleavage upstream (Figures 7 and 8). Thus, RNase E is able to reach more than one ‘handhold’ (i.e. unpaired region) while retaining hold of another. We also found that cleavage at the +447 site in *rne* mRNA could be enhanced by a region on either its 5′ or 3′ side (Figure 9). Therefore, RNase E can also reach the same ‘handhold’ from more than one position. Such flexibility may explain why simply increasing the single-stranded character of an otherwise 5′ monophosphate-dependent substrate was sufficient to negate the requirement for 5′ sensing (26). That is to say, access to an additional handhold(s) removed the requirement for a ‘foothold’ (i.e. 5′-monophosphorylated end). Previous work has shown that RNase E cleavage can be ‘shifted’ by chemical modification of sites in an oligonucleotide substrate (64). In addition, we envisage that one or more handholds can be part of a folded structure and that should the most 3′ of two unpaired regions used as handholds be cleaved first, cleavage of the most 5′ region could be facilitated in a second step by RNase E using another unpaired region as a handhold or perhaps a 5′-monophosphorylated end as a foothold. Which of the unpaired regions used in binding is cleaved preferentially is likely to be determined, at least in part, by their sequences (65,66). The above modes of RNase E cleavage are shown schematically (Figure 10).

### The evolution of 5′-end sensing

The finding that the simultaneous binding to two or perhaps more unpaired regions enables direct entry provides a simple basis for understanding the evolution of 5′ sensing (8). Without a pocket that binds 5′-monophosphorylated ends, cleavage of an upstream region would hamper the subsequent cleavage of a downstream region(s) as a result of weakening the overall interaction (i.e. the lost handhold could not become a foothold). This scenario is exemplified by the E5 site in the *argX-hisR-leuT-proM* tRNA precursor, which can only be cleaved efficiently after cleavage of an upstream region, provided the 5′ sensor is functional (13). The evolution of the sensor would have been relatively straightforward given few amino acids actually contact 5′-monophosphorylated ends (for details, see ‘Introduction’ section). The evolution of a pyrophosphohydrolase with activity against RNA in a subsequent event would have permitted interaction with the 5′ portions of nascent transcripts that otherwise could have been inaccessible to RNase E. The driver for this step could have been an increase in the efficiency of a processing or degradation step(s) that was growth limiting. Simultaneous access to multiple unpaired regions may otherwise have been impeded by RNA folding or the association of proteins or both (12).

### The role of the degradosome

The work described here also adds further examples to a growing list of cleavages both 5′-end dependent and independent that can be reconstituted *in vitro* using only the NTH of RNase E (12–14,26–27). However, deletion of the C-terminal half of RNase E is known to impede the degradation of at least some mRNAs *in vivo* (23–24,67–68). One possible explanation is that the association of the catalytic domain of RNase E with the degradosome on the inner surface of the cytoplasmic membrane facilitates the rapid removal of RNase E cleavage products (e.g. mRNA decay intermediates). This is likely to be functionally important as we have found *in vitro* that the products of the cleavage of some transcripts can remain tightly associated with RNase E and inhibit further rounds of cleavage (Kime, Clarke and McDowall, unpublished data). Moreover, it has been reported that RNases in addition to components of the degradosome are membrane associated (69). An increasing amount of evidence points to the spatial organisation of steps in gene expression including mRNA decay (70).

We are currently investigating the extent to which the ability of cleavage products to inhibit additional rounds of cleavage by RNase E is dependent on a functional 5′ sensor. The ability of WT RNase E to bind the 5′ ends of downstream cleavage products may explain why NTH-RNase E is less efficient than its T170V counterpart at cleaving an excess of substrate that is 5′ hydroxylated (Figure 1, panel C). Enhanced cleavage by NTH-RNase E at other sites would explain why this is not obvious when the substrate is 5′ monophosphorylated (Figure 1, panel B). Interestingly, the CTH of RNase E is not indispensable in combination with mutations that disable 5′ sensing by RNase E (14,27) or disrupt the RppH pyrophosphohydrolase (71). An explanation consistent with these findings is that the CTH may enable sufficient levels of a critical processing or degradation event(s), normally mediated via an interaction with a 5′-monophosphorylated end generated by RppH, to be mediated by direct entry. This interpretation does not exclude the possibility that the CTH also enhances 5′ end-dependent cleavage by RNase E.

### Interpretation of the RNA-seq data

While our approach of combining RNA-seq data for *in vivo* and *in vitro* comparisons has been successful in identifying sites of efficient direct entry (Figures 5–9), the analysis is complicated by the fact that (i) the detection of substantial levels of cleavage *in vitro* requires that the substrate is present at sufficient levels *in vivo* against a background of active processing and degradation and (ii) not all of the downstream species of RNase E cleavage *in vivo* will necessarily be depleted substantially following inactivation of the temperature-sensitive mutant, some will be substrates for further cleavage by RNase E. For example, although direct entry cleavage at RNase E-dependent sites within the 3′ end of *epd* and 5′ end of *pgk* (46) and on the 3′ sides of *argX* and *leuT* tRNA has been demonstrated *in vitro* using 5′-triphosphorylated substrates (12,13), in each case one of the *M*-values for the *in vivo* or *in vitro* comparison was substantially lower than the other. Thus, there is value in following up cleavages for which there is strong evidence *in vivo* or *in vitro*, but not both.

Moreover, analysis of the *in vivo* RNA-seq data on its own is providing new insight into the roles of RNase E and G, some of which will be 5′-end dependent. For example, we have found that a cleavage that occurs during exponential growth and removes the anti-Shine-Dalgarno sequence at the 3′ end of 16S rRNA, generating a downstream product with a 5′-monophosphorylated end (Romero *et al.*, submitted) is highly RNase E dependent (*M*-value of 5.1 at position 1507 within the 16S rRNA of the *rrnE* operon). We have also identified for RNase G over 80 instances, in addition to *adhE* and *eno mRNA*, where the inactivation of this enzyme leads to the accumulation of a 5′-monophosphorylated transcript extended by <200 nucleotides from *rng*-dependent sites (data not shown). This suggests a wide-spread role for RNase G in the removal of 5′ sequences. Moreover, the 5′ ends of five of the extended species that accumulate correspond to the transcription start sites of *aceE*, *acpP*, *gnsB*, *slyD* and *ynhf* mRNA (Romero *et al.*, submitted), indicating that as found for RNase E, a subset of cleavages by RNase G are likely dependent on pyrophosphate removal.

### Possible new roles emerging from the RNA-seq analysis

Finally, we note with interest that cleavage within the evolutionarily conserved helix/loop 70 of 23S rRNA, which forms the active centre of the ribosome (for review, see (54)), has recently been reported for *Mycobacterium tuberculosis*. However, the enzyme responsible in this organism is not RNase E, but members of the MazF family (72,73). Members of the MazF family have also been shown to cleave towards the 3′ end of 16S RNA resulting in the removal of the anti-Shine Dalgarno sequence in *E. coli* (74) as well as *M. tuberculosis* (73). Whether a member of the MazF family also cleaves in the conserved helix/loop 70 of 23S rRNA in *E. coli* has not been determined to our knowledge, but would seem likely. The identification in this study of RNase E cleavage both *in vitro* and *in vivo* within the helix/loop 70 of 23S rRNA of *E. coli* (*M*-values of 10.9 and 6.0 in the *rrnG* operon, respectively; see Supplementary Table S2) suggests, as reported initially for a MazF-family member (72,73), a role for RNase E in the control of global translational activity via a mechanism that does not involve mRNA cleavage. As indicated above, RNase E may also have a role in determining the proportion of ribosomes that contain the anti-Shine-Dalgarno sequence. In this and another study (Romero *et al.*, submitted), cleavage of 16S rRNA *in vivo* was detected at the same location reported for *E. coli* MazF (74). The 5′-hydoxylated products of cleavage by members of the MazF family would not have been detected by our RNA-seq approach (36). It should be noted that *E. coli* does not appear to contain an RNA 5′ kinase, or at least one that can efficiently phosphorylate a 5′-hydroxylated end generated *in vivo* using a hammerhead ribozyme (Kime and McDowall, unpublished data). Together, the above results indicate that more than one ribonuclease family may control the activity or specificity or both of ribosomes via processing of their RNA backbones.

## SUMMARY

The application of RNA-seq to the study of *E. coli* RNase E has provided experimental evidence that direct entry represents a major route by which this enzyme can mediate steps central to RNA processing and degradation. It has also identified potential new roles for RNase E in for example the control of ribosome activity and specificity. More generally, our RNA-seq approach provides an improved platform to study events controlling the lifecycles of RNA and to identify substrates that can be characterized and compared to discover the underlying structural molecular biology. For example the analysis of additional direct-entry substrates reported here is consistent with a model in which the binding of RNase E is facilitated by simultaneous interaction with multiple unpaired regions. These simple requirements may maximise the rate of RNA degradation by permitting multiple sites to be surveyed directly without being constrained by 5′-end tethering. This ability is likely to be important not only for the rapid degradation of mRNAs whose translation is poor or blocked, but in ensuring that the only rRNAs that persist are those that are folded correctly and associated with the full complement of r-proteins to form functional ribosomal subunits.

## SUPPLEMENTARY DATA

Supplementary Data are available at NAR Online.

SUPPLEMENTARY DATA

## ACCESSION NUMBERS

The RNA-seq data has been deposited in the Gene Expression Omnibus (GEO) repository (GEO accession number GSE58285) and NCBI accession number U00096.2.
