# Supplementary material for: Direct entry by RNase E is a major pathway for the degradation and processing of RNA in Escherichia coli
Source: Nucleic Acids Res. 2014 Sep 18;42(18):11733–51. doi: 10.1093/nar/gku808 (PMC4191395; doi:10.1093/nar/gku808)
Supplement: SUPPLEMENTARY DATA [file supp_42_18_11733__index.html]

Direct entry by RNase E is a major pathway for the degradation and processing of RNA in Escherichia coli — SUPPLEMENTARY DATA 

# Direct entry by RNase E is a major pathway for the degradation and processing of RNA in *Escherichia coli*

## SUPPLEMENTARY DATA

**Files in this Data Supplement:**

- SUPPLEMENTARY DATA
