# Supplementary material for: Direct entry by RNase E is a major pathway for the degradation and processing of RNA in Escherichia coli
Source: Nucleic Acids Res. 2014 Sep 18;42(18):11733–51. doi: 10.1093/nar/gku808 (PMC4191395; doi:10.1093/nar/gku808)
Supplement: SUPPLEMENTARY DATA [file supp_gku808_nar-01693-h-2014-File012.pdf]

Supplementary Material

Direct entry by RNase E is a major pathway for the degradation and processing of RNA in *E. coli*

Justin E. Clarke[+], Louise Kime[+], David Romero A., and Kenneth J. McDowall*

[*]Corresponding author: Kenneth J. McDowall (k.j.mcdowall@leeds.ac.uk)

[+]Joint first authors

Astbury Centre for Structural Molecular Biology, School of Molecular and Cellular Biology, Faculty of Biological Sciences, University of Leeds, Leeds, LS2 9JT, UK

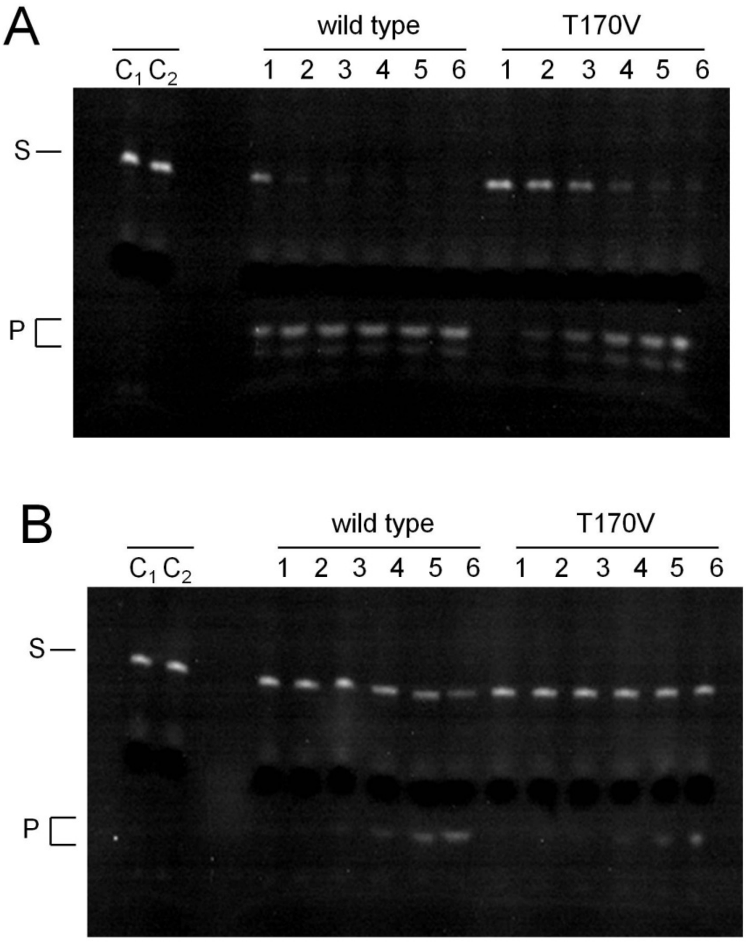

FIG S1. Cleavage of derivatives of BR13 by NTH-RNase E in the presence of *E. coli* total RNA. A 5'-monophosphorylated (A) or 5'-hydroxylated (B) derivative of BR13 (1), named LU13, with GAG rather than GGG at the 5' end (2) was pre-incubated with *E. coli* total RNA before addition of enzyme. Both substrates were labelled with fluorescein at the 3' end. BR13 is an oligonucleotide substrate derived from the RNase E-cleaved region at the 5' end of the antisense regulator of ColE1-type plasmid replication. The concentrations of NTH-RNase E, LU13 and total RNA at the start of the reactions were 300 nM, 15 nM and 30 ng/µl, respectively. Lanes 1-6 contain samples taken 0, 2, 5, 15, 30 and 60 min after mixing of substrate and enzyme. Lanes C_1 and C_2 contain substrate incubated without enzyme for 0 and 60 min, respectively. Labelling on the left of each panel indicates the position of substrate (S) and products (P).

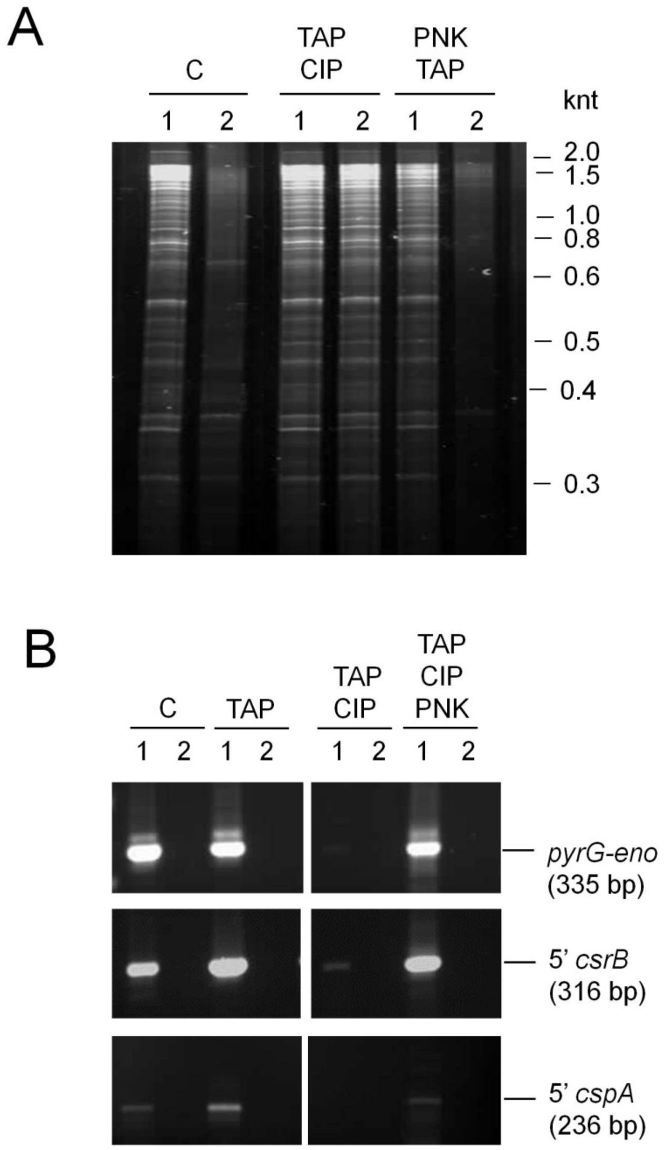

FIG S2. Confirming the 5' status of RNA samples prior to incubation with RNase E. (A) Analysis of enriched mRNA using Terminator™ 5' end-dependent exonuclease. Three samples of enriched mRNA were analysed: untreated (C); treated with tobacco acid pyrophosphatase (TAP) followed by calf intestinal phosphatase (CIP) to generate 5'-hydroxylated ends; or treated with T4 polynucleotide kinase (PNK) followed by TAP to generate 5'-monophosphorylated ends. The RNA was then incubated in the absence (Lane 1) or presence (Lane 2) of Terminator™ exonuclease, which is a 5'-3' exonuclease that at low concentrations is specific for 5'-monophosphorylated RNA (3). The products were analysed by denaturing gel electrophoresis as described in Fig. 1. The positions of RNA size markers (not shown) are indicated on the right. For details of each of the treatments and digestion by Terminator™ exonuclease, see 'Materials and Methods'. The combination of TAP and CIP produced RNA that was resistant to TEX indicating that it had been 5' hydroxylated as expected. While bulk RNA was already susceptible to TEX prior to treatment, the combination of PNK and TAP increased the susceptibility further as evidenced by the reduction in the amount of RNA species < 1 knt. This indicated that the RNA had been 5' monophosphorylated. (B) Analysis of total RNA using RNA ligase-mediated RT-

PCR. This technique allows the detection of RNAs that are 5' monophosphorylated. Total RNA was treated with the indicated enzymes to modify the 5' ends. The lanes labelled C represent RNA that has not been treated with 5' end modifying enzymes. Following ligation of an RNA adapter to the 5' ends of the total RNA, the RNA was reverse transcribed in the presence (Lane 1) or absence (Lane 2) of reverse transcriptase. PCR was then used to analyse the 5'-end status of targets using a forward primer specific for the RNA adapter and a reverse primer specific for the amplification of an RNase E-dependent processing site in the intergenic region between *pyrG* and *eno* and the transcription start sites of *csrB* and *cspA*. Labelling on the right identifies the targets and the expected sizes in base pairs of the amplicons. The observed mobility of these products relative to size markers was consistent with their expected size (data not shown). The RLM-PCR analysis was done as described previously (4). The sequence of the primer specific for the RNA adapter was 5'-CGAAGACAACAAAGAAGTTCAACTC and the sequences of the primers specific for *pyrG-eno*, *csrB* and *cspA* were 5'-GTCAATGCCAGCCTGATCTT, 5'-CAACTTTTCCTCTGGCCTTG and 5'-TGTCAGCGTTGAACCATTTT, respectively. As expected, TAP treatment increased the amount of *csrB* and *cspA* mRNA that was amplified, while not affecting the amplification of the *pyrG-eno* intermediate, and subsequent CIP treatment blocked amplification of all three species. Finally, treatment with PNK restored amplification to maximum levels. Amplification of *csrB* and *cspA* mRNA in the absence of TAP treatment was expected and reflects 'decapping' *in vivo*. Thus, all three enzymes appeared to modify RNA as expected. The efficiency of 5' phosphate removal for some RNAs was increased by incubating with TAP followed by CIP rather than with CIP alone.

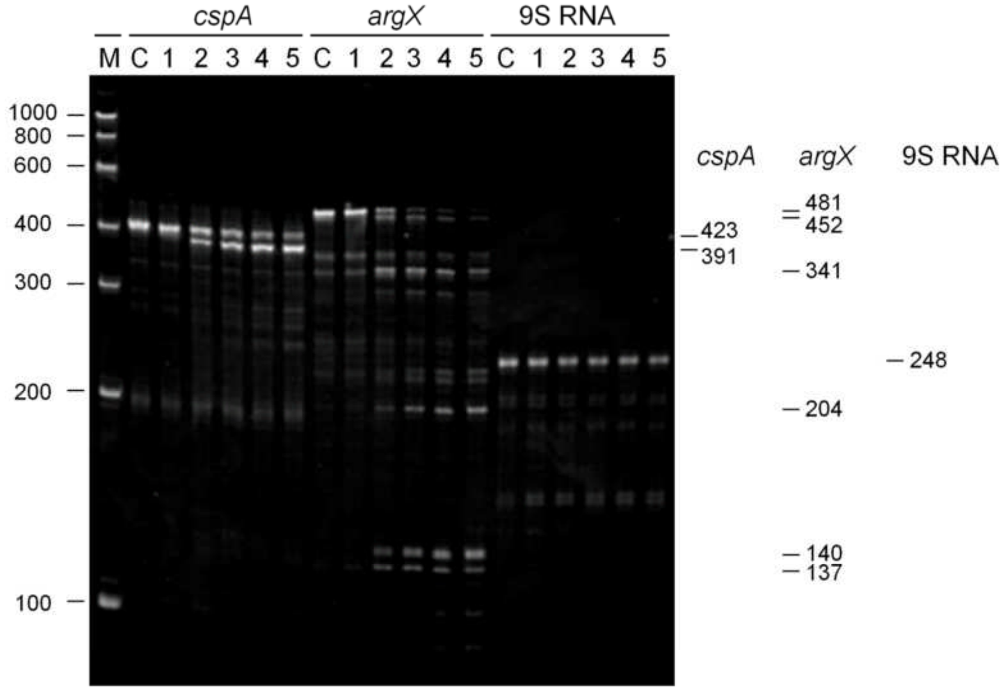

FIG S3. Positive and negative controls for the assay of direct-entry cleavage. The *cspA* mRNA and *argX-hisR-leuT-proM* precursor provide positive controls, while 9S RNA provides a negative control. The 5'-triphosphorylated transcripts were generated by *in vitro* transcription using templates and conditions described previously (3,4). The conditions for the cleavage assays, the preparation of the T170V mutant of RNase E, and the analysis of reaction products by denaturing gel electrophoresis were also as described previously (3,4). The enzyme monomer and initial substrate concentrations at the start of each reaction were 20 and 180 nM, respectively. The RNA was stained using ethidium bromide. Lanes 1–5 contain samples taken 0, 5, 15, 30, and 60 min, respectively, after mixing substrate and enzyme. Lane C contains substrate incubated without enzyme for 60 min. The sizes (nt) of RNA markers (Thermo Scientific RiboRuler Low Range) are indicated on the left of the panel. The sizes of the major species are indicated on the right of the panel. The *cspA* species of 391 nt corresponds to the upstream product of cleavage on the 5' side of the transcription terminator (2,5). The *argX* species of 452, 341, 204, 140 and 137 nt correspond to the 5' end to E5 site, E1 to E5, E1 to E3, 5' end to E1, and E3 to 3' end, respectively (3).

Transcript	Primer	Primer sequence (5' to 3')
rnc (871 nt)	FWD	ATCCTAATACGACTCACTATAGGGGAAGTTTAAGGTTGGCAC
rnc (358 nt)	FWD	ATCCTAATACGACTCACTATAGGGATTAATTGGTGGCGTA
	RVS	TAAATCCGCAGTAACTTTTATCG
uspF	FWD	ATCCTAATACGACTCACTATAGGGGGATACTAAAACAGG
	RVS	AAAGCCCGCAGCAATGTG
tomB/hha	FWD	ATCCTAATACGACTCACTATAGGGCTAACAAACGAGAGGGCAAG
	RVS	TCTTAATAAACAGCCGGTTATAGC
fdhE	FWD	ATCCTAATACGACTCACTATAGGGGCATATCTACGCCGCCCTTT
	RVS	TTAATGCGCGCAGTCAGATC
cspA	FWD	ATCCTAATACGACTCACTATAGGGTTTGACGTACAGACC
	RVS	AAAATCCCCGCCAAATGGCAGGG
9S (*rrfB*)	FWD	ATCCTAATACGACTCACTATAGGGAAGCTGTTTTGGCGGATGAG
	RVS	ACGAAAGGCCCAGTCTTTC
argX	FWD	ATCCTAATACGACTCACTATAGGGAACGGCGCTAAGCGCCCG
	RVS	AAAAAACCCCGCCGAAGCGG
rne (1138 nt)	FWD	ATCCTAATACGACTCACTATAGGGGTTTCCGTGTCCATCCTTGT
	RVS	CAGTTTGATTTTGCTGCTGA
rne (457 nt)	FWD	ATCCTAATACGACTCACTATAGGGAATCACCCGCATTGAACC
	RVS	TTTTTTGATGGCTTCCCAGTG
rne (409 nt)	FWD	ATCCTAATACGACTCACTATAGGGAATCACCCGCATTGAACC
	RVS	CAGCGCCTCAGCAGATTT
rne (315 nt)	FWD	ATCCTAATACGACTCACTATAGGGGTGTTGCGTGAAGGTCAGG
	RVS	TTTTTTGATGGCTTCCCAGTG
rne (267 nt)	FWD	ATCCTAATACGACTCACTATAGGGGTGTTGCGTGAAGGTCAGG
	RVS	CAGCGCCTCAGCAGATTT
cspC	FWD	ATCCTAATACGACTCACTATAGGGACGCCAGTTTAAGTATCTGCC
	RVS	TTCACGCGAAAGAGGCT
uspG	FWD	ATCCTAATACGACTCACTATAGGGGCGAATGATTTGTTCATGATT
	RVS	ATTAAAAAGCCCCGCCG
ftsI	FWD	ATCCTAATACGACTCACTATAGGGTGGCGCTACCAGGCG
	RVS	TTACGATCTGCCACCTGTCCT
envZ	FWD	ATCCTAATACGACTCACTATAGGGATGTTGATGACCGACAAACTG
	RVS	GGCCGCCCAGTTGCT
glnW-metU-glnV	FWD	ATCCTAATACGACTCACTATAGGGTGGGGTATCGCCAAGC
	RVS	TGGCTGGGGTACGAGGAT
glyX-glyY	FWD	ATCCTAATACGACTCACTATAGGGGCGGGAATAGCTCAGTTGG
	RVS	GCGTCGCTGTGGATATTTTATT

Table S1. The sequence of primers used to generate cDNA templates for T7 *in vitro* transcription. Underlining indicates the position of the sequence of the T7 polymerase promoter in each of the forward primers. The numbers in parentheses indicate the sizes of the corresponding transcripts.

Position	Strand	M (*in vivo*)	M (*in vitro*)	Associated Gene	Position
93136	fwd	7.17	9.96	*ftsI*	internal
93144	fwd	6.40	8.24	*ftsI*	internal
113122	rvs	5.46	8.75	*coaE*	internal
190802	fwd	5.15	9.42	*tsf*	5' intergenic
227695	fwd	5.43	9.61	23S rRNA of *rrnH*	internal
323669	fwd	5.90	9.49	*ykgG*	internal
447668	rvs	5.61	8.74	*cyoC*	internal
449135	rvs	6.69	8.43	*cyoB*	internal
449152	rvs	5.70	8.31	*cyoB*	internal
452839	rvs	8.03	11.82	*yajG*	internal
479558	rvs	5.19	9.91	*ybaJ*	internal
640655	rvs	5.42	8.21	*uspG*	3' UTR
709635	rvs	5.29	9.11	*fur*	internal
754611	fwd	9.77	8.09	*sdhC*	internal
754977	fwd	5.29	8.22	*sdhD*	internal
754978	fwd	5.53	8.57	*sdhD*	internal
755103	fwd	5.04	9.66	*sdhD*	internal
755105	fwd	5.49	10.75	*sdhD*	internal
778815	fwd	6.30	8.82	*ybgF*	5' intergenic
846518	rvs	5.37	8.58	*glnH*	internal
963323	fwd	5.46	8.48	*ihfB*	internal
985413	rvs	6.29	10.39	*ompF*	internal
985851	rvs	5.36	8.54	*ompF*	internal
985860	rvs	5.67	9.84	*ompF*	internal
1018886	rvs	5.32	9.63	*ompA*	internal
1143045	rvs	5.00	8.08	*rne*	internal
1224121	rvs	7.24	8.65	*minD*	internal
1224577	rvs	5.36	7.99	*minD*	internal
1300973	fwd	6.09	8.37	*oppB*	internal
1300979	fwd	5.73	9.11	*oppB*	internal
1407235	fwd	6.19	8.01	FnrS sRNA	internal
1433290	rvs	5.69	8.68	*uspF*	internal
1487678	fwd	5.29	9.08	*aldA*	internal
1487690	fwd	6.37	10.32	*aldA*	internal
1515133	rvs	6.36	8.08	*yncL*	internal
1900641	fwd	5.29	10.13	*manX*	internal
1905419	fwd	5.39	10.30	unknown	
1905439	rvs	5.67	8.94	*cspC*	internal
1905470	rvs	7.42	9.07	*cspC*	5' intergenic
2109053	rvs	6.99	8.58	*rfbD*	5' intergenic
2170205	rvs	5.44	8.23	*gatD*	internal
2170543	rvs	6.27	9.40	*gatD*	internal
2170904	rvs	5.04	8.76	*gatD*	5' intergenic
2170945	rvs	5.29	9.66	*gatC*	internal
2170946	rvs	6.22	8.58	*gatC*	internal
2170947	rvs	6.50	8.67	*gatC*	internal
2170950	rvs	5.64	9.76	*gatC*	internal
2171642	rvs	7.65	8.39	*gatC*	internal
2171832	rvs	6.41	9.82	*gatC*	internal
2173243	rvs	6.38	8.89	*gatZ*	internal
2237371	rvs	6.02	8.76	*mglB*	internal
2238426	fwd	5.67	8.45	unknown	
2238477	rvs	6.75	8.67	*mglB*	5' intergenic

2268103	fwd	7.20	9.04	*mepS (spr)*	internal
2388534	rvs	8.01	8.64	*nuoN*	internal
2392843	rvs	6.86	8.41	*nuoL*	internal
2393046	rvs	5.67	8.73	*nuoL*	internal
2397080	rvs	8.04	8.34	*nuoG*	internal
2397802	rvs	5.36	8.75	*nuoG*	internal
2397961	rvs	8.04	8.99	*nuoG*	internal
2401345	rvs	5.95	8.26	*nuoC*	internal
2402226	rvs	5.81	9.08	*nuoB*	internal
2402633	rvs	6.41	10.08	*nuoB*	internal
2403043	rvs	5.04	8.89	*nuoA*	internal
2519165	fwd	6.43	8.85	*valX* tRNA	3' intergenic
2576334	rvs	5.58	8.37	*maeB*	internal
2701527	rvs	5.49	9.17	*rnc*	internal
2721316	fwd	5.43	8.76	*pssA*	internal
2725271	rvs	6.04	10.94	23S rRNA of *rrnG*	internal
2743349	fwd	5.78	10.78	unknown	
2743400	rvs	5.09	11.27	*rimM*	internal
2865691	rvs	5.42	8.28	*nlpD*	internal
2865699	rvs	5.83	8.52	*nlpD*	internal
2922302	rvs	6.24	9.22	*csrB*	internal
3144295	rvs	6.02	8.89	*hybO*	5' UTR
3331735	fwd	5.32	8.19	*ispB*	internal
3422870	rvs	5.93	9.98	23S rRNA of *rrnD*	internal
3430051	rvs	6.36	8.17	*smg*	internal
3440310	rvs	5.70	8.96	*rpsM*	internal
3533575	rvs	5.13	8.17	*envZ*	internal
3551863	fwd	6.09	9.40	*malT*	internal
3887308	fwd	6.60	8.93	*tnaA*	internal
3887483	fwd	7.69	8.05	*tnaA*	internal
3888162	fwd	5.23	8.66	*tnaA*	internal
3888164	fwd	5.64	9.47	*tnaA*	internal
3888212	rvs	5.01	8.84	unknown	
3888213	rvs	6.17	9.76	unknown	
3888215	fwd	5.46	8.07	*tnaB*	5' intergenic
3888613	fwd	6.00	8.31	*tnaB*	internal
3934182	fwd	6.00	8.51	*rbsC*	internal
3980610	fwd	9.01	9.13	*hisR* tRNA	3' intergenic
3980835	fwd	6.30	9.67	*proM* tRNA	3' intergenic
4079159	rvs	5.17	10.13	*fdhE*	internal
4130357	fwd	5.73	8.47	*metL*	3' UTR
4379660	rvs	5.73	8.03	*frdA*	internal
4390571	fwd	5.46	8.43	*glyX* tRNA	3' intergenic
4390572	fwd	5.93	10.39	*glyX* tRNA	3' intergenic
4414751	rvs	5.81	8.31	*yjfO*	internal
4437515	fwd	5.09	8.30	*ytfK*	5' UTR

Table S2. The top 100 sites of RNase E direct entry *in vitro*. Sites with the highest M values upon incubation with T170V after 10 min were selected from those which also had a minimum M value of 5 upon inactivation of *rne in vivo*. The chromosomal positions and strand were used to map these sites to their associated genes. The position of cleavage relative to the gene coding region is also indicated.

References

1. McDowall, K.J., Kaberdin, V.R., Wu, S.W., Cohen, S.N. and Lin-Chao, S. (1995) Site-specific RNase E cleavage of oligonucleotides and inhibition by stem-loops. *Nature*, **374**, 287-290.
2. Kime, L., Jourdan, S.S., Stead, J.A., Hidalgo-Sastre, A. and McDowall, K.J. (2010) Rapid cleavage of RNA by RNase E in the absence of 5' monophosphate stimulation. *Mol. Microbiol.*, **76**, 590-604.
3. Kime, L., Clarke, J.E., Romero, A.D., Grasby, J.A. and McDowall, K.J. (2014) Adjacent single-stranded regions mediate processing of tRNA precursors by RNase E direct entry. *Nucleic Acids Res.*, **42**, 4577-4589.
4. Kime, L., Jourdan, S.S. and McDowall, K.J. (2008) In Maquat, L. E. and Arraiano, C. M. (eds.), *Methods in Enzymology: RNA Turnover in Bacteria, Archaea and Organelles*. Academic Press, Vol. 447, pp. 215-241.
5. Hankins, J.S., Zappavigna, C., Prud'homme-Genereux, A. and Mackie, G.A. (2007) Role of RNA structure and susceptibility to RNase E in regulation of a cold shock mRNA, *cspA* mRNA. *J. Bacteriol.*, **189**, 4353-4358.
